# Migratory wild birds carrying multidrug-resistant *Escherichia coli* as potential transmitters of antimicrobial resistance in China

**DOI:** 10.1371/journal.pone.0261444

**Published:** 2021-12-15

**Authors:** Yue Yuan, Bing Liang, Bo-wen Jiang, Ling-wei Zhu, Tie-cheng Wang, Yuan-guo Li, Jun Liu, Xue-jun Guo, Xue Ji, Yang Sun

**Affiliations:** 1 Engineering Research Center of Glycoconjugates, Ministry of Education, School of Life Sciences, Northeast Normal University, Changchun, China; 2 Changchun Veterinary Research Institute, Chinese Academy of Agricultural Sciences, Changchun, China; 3 Key Laboratory of Jilin Province for Zoonosis Prevention and Control, Changchun, China; Suez Canal University, EGYPT

## Abstract

Migratory birds play an important role in the spread of multidrug-resistant (MDR) bacteria. To investigate the prevalence of MDR *Escherichia coli* in migratory birds in China and potential relationships with the environment, a total of 1387 samples (fecal samples, cloacal swabs, or throat swabs) were collected from migratory birds from three different river basins in China. The collected samples were processed and subjected to bacteriological examinations. Antimicrobial susceptibility testing of the recovered isolates was performed using the E-test for the detection of minimum inhibitory concentrations (MICs). Some antibiotic resistance genes were detected and the PCR products were confirmed by sequencing. In total, 478 (34.7%) *E*. *coli* isolates were recovered. The results showed that the drug-resistant *E*. *coli* isolates were highly resistant to β-lactams (43.7%) and tetracycline (22.6%), and 73 (15.3%) were MDR, including eight that were extended spectrum β-lactamase-positive. The retrieved strains harbored the *bla*_CTX-M_, *bla*_TEM-1_, *tet(*A*)*, *tet(*B*)*, *tet(*M*)*, *sul1*, *sul2*, *sul3*, *cmlA*, *floR*, and *intI1* genes with a prevalence of 5.9%, 36.4%, 80.5%, 11.9%, 6.8%, 6.8%, 47.5%, 12.7%, 50.8%, 37.3%, and 61.0%, respectively. The drug resistance rate of the isolates from southern China was higher than those from northern China. The *E*. *coli* samples collected for migratory birds in the Pearl River Basin had the highest proportion (46.7%) MDR isolates. Furthermore, MDR bacteria carried by migratory birds were closely related to the antibiotic content in the basin, which confirms that MDR bacteria carried by migratory birds are likely acquired from the environment. This study also confirmed that migratory birds are potential transmitters of MDR bacteria, demonstrating the need to reduce the use and emission of antibiotics and further in-depth studies on the mechanisms underlying drug resistance of bacteria.

## Introduction

Antimicrobial resistance (AMR) is among the most important threats to public health. Inadequate treatment of waste from humans and livestock containing antimicrobial drugs leads to the environmental dissemination of antibiotic-resistant bacteria. The resulting spread of multidrug-resistant (MDR) bacteria and antibiotic resistance genes (ARGs) poses a significant threat to the health of humans and animals worldwide [1,2]. The prevalence of MDR bacteria continues to increase worldwide. Several recent investigations reported the emergence of MDR bacterial pathogens from different origins, including humans, birds, cattle, and fish, which increase the need for routine antimicrobial susceptibility testing to choose an appropriate antibiotic as well as the screening of the emerging MDR strains.

*Escherichia coli* is one of the best bacterial models to study the spread of AMR [3,4]. Most *E*. *coli* strains that reside in the intestines are harmless, but some can cause severe diarrhea. Some *E*. *coli* obtain a series of functional genes through horizontal transfer, which allow for colonization of the host intestine. The *E*. *coli* strains that cause diarrhea include the enterotoxigenic, enterohemorrhagic, enteroinvasive, enteropathogenic, enteroaggregative, diffusely adherent, and cell-detaching pathotypes [5]. The pathogenic mechanisms of the *E*. *coli* pathotypes differ. For example, enterotoxigenic *E*. *coli*, which is characterized by the production of colonization factors and at least one type of heat-labile (LT) or heat-stable (ST) enterotoxin, is also an important pathogen in other domesticated animals, including pigs and cattle [6], while enteropathogenic *E*. *coli* does not produce LT and ST enterotoxins, but rather generates attaching and effacing lesions to the intestinal epithelium [6]. Recent studies have reported the emergence of MDR pathogens [7–9]. β-lactam drugs account for about 60% of all prescribed antibacterial agents, probably due to safety and adequacy. However, overuse of these agents had led to the rapid emergence of MDR pathogens [10]. The frequent occurrence of MDR bacteria indicates the excessive and arbitrary use of antibiotics, which poses a great threat to public health, and reflects the necessity for the development of new potent and safe antibiotics [11].

Wild animals, especially those that migrate, have a great influence on the spread of MDR bacteria and ARGs [12]. Due to the diversity in ecological niches, migratory birds act as reservoirs and transporters of antibiotic-resistant bacteria and consequently play a significant epidemiological role in the dissemination of ARGs [13,14]. Globally, there are eight bird migratory routes, which are distributed among all continents, including Antarctica. Migratory birds, which are abundant in number and have a wide range of activities, carry foreign ARGs during migration that facilitates the dissemination of MDR bacteria and ARGs in the environment [15–18]. Three of the eight bird migratory routes pass through China, including the East Asian-Australasian Flyway, which has the greatest diversity and populations of migratory birds [19]. Many migratory birds choose wetlands around rivers and lakes along this route as habitats for breeding or wintering. Some studies have investigated the intercontinental transmission of MDR bacteria and ARGs by migratory birds to different areas of China [18,20,21]. However, no study has yet to comprehensively assess MDR bacteria isolated from migratory birds in different regions and river basins in China.

Therefore, in order to better understand the dissemination of MDR bacteria and ARGs by migratory birds along the East Asian-Australasian Flyway and potential environmental impacts, more than 1000 samples were collected from migratory birds in different regions in China. The resistant phenotypes and ARGs of *E*. *coli* isolated from these samples were identified and the drug resistance rates of the isolates from different river basins were compared. By investigating the relationship between the MDR *E*. *coli* carried by migratory birds and environmental factors, especially antibiotic discharge, the effects of bird migration on the transmission of MDR bacteria and ARGs were assessed.

## Materials and methods

### Sample collection

**Fig 1:**
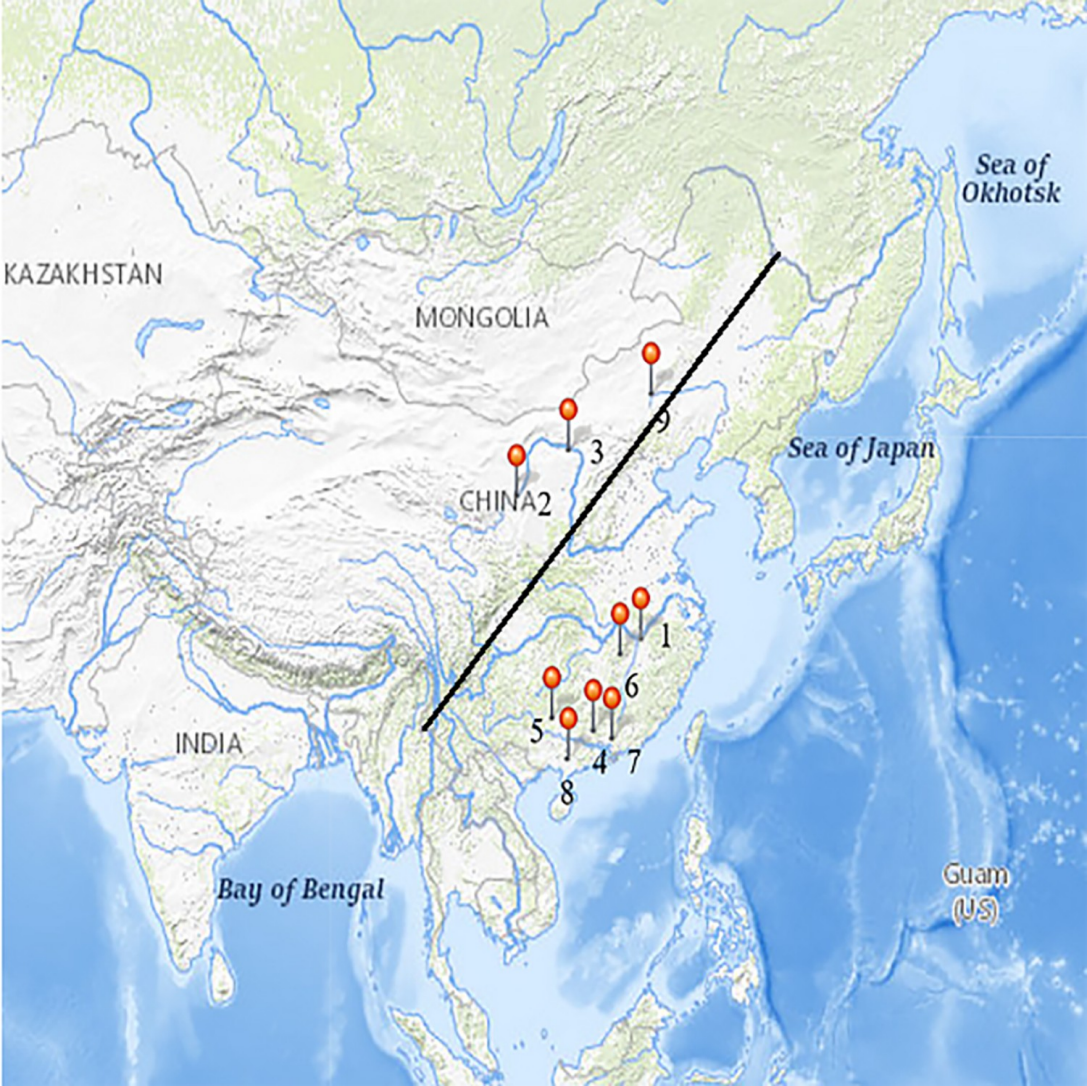
The sampling sites used in this study. Note. The black line on the map represents the “Aihui-Tengchong Line”. The stars indicate different sampling locations and the numbers in the upper right corner are sorted by sampling time. Sampling locations: 1. Poyang Lake, Jiangxi Province (28°N, 116°E). 2. Ningxia Hui Autonomous Region (37°N, 105°E). 3. Honghaizi Wetland Park, Inner Mongolia;/ Ordos City, Inner Mongolia (39°N, 109°E). 4. Zhaoqing, Guangdong Province (23°N, 112°E). 5. Nanning, Guangxi Province (22°N, 108°E). 6. Suichuan, Jiangxi Province (26°N, 114°E). 7. Shenzhen, Guangdong Province (22°N, 114°E). 8. Zhanjiang, Guangdong Province (21°N, 109°E). 9. Dali Lake, Inner Mongolia(42°N, 115°E).

All migratory bird samples were collected from May 2017 to June 2019 from six provinces in China (Fig 1, obtained from the USGS National Map Viewer). The procedures for handling and sampling of migratory birds were approved by the State Forestry Administration and the Laboratory Animal Welfare and Ethics Committee of the Changchun Veterinary Research Institute, Chinese Academy of Agricultural Sciences (AMMS-11-2020-11), and conducted in accordance with the Guidelines for the Care and Use of Animals in Research. No anesthesia, euthanasia, or animal sacrifice was conducted in this study. The sampling provinces located in Northeast China, Northwest China, Southern China and other regions were all within the range of the East Asian-Australasian Flyway. Feces, cloacal swabs, and throat swabs were collected under the supervision of the Wild Animal Sources and Diseases Inspection Station, National Forestry and Grassland Bureau of China. All precautions were made to avoid any potential harm to the birds. The swabs were stored in physiological saline containing 20% glycerol at -80°C for a short period and transported to our laboratory on dry ice.

### Isolation and identification of *E*. *coli*

The samples (n = 1387) were resuspended in physiological saline, plated on MacConkey ager (BD Biosciences, San Jose, CA, USA), and incubated overnight at 37°C. One suspected *E*. *coli* colony was selected from each plate and re-cultured on McConkey agar for subsequent analysis. The identification and antimicrobial susceptibility of presumptive *E*. *coli* isolates were determined using the NMIC/ID 4 panel of the BD Phoenix™ Automated Identification and Susceptibility Testing System (Becton, Dickinson and Company, Franklin Lakes, NJ, USA) [22]. Bacterial strains were preliminarily classified as extended spectrum beta-lactamase (ESBL)-producers by the ESBL screen flow application of the same system. The isolates found to be resistant to at least three different classes of antimicrobial agents were classified as MDR bacteria [23].

### Detection of minimum inhibitory concentration (MIC)

The MIC of *E*. *coli* isolates with the drug resistant phenotype was tested on Mueller–Hinton agar (bioMérieux, Marcy-l’Étoile, France) plates using commercially available E-test strips (Liofilchem SRL, Roseto degli Abruzzi, Italy) containing different types of antibiotics. Antimicrobial susceptibility testing include aminoglycosides (amikacin and gentamicin), β-lactams (cefazolin, cefotaxime, cefepime, aztreonam, ampicillin, piperacillin, amoxicillin/clavulanic and ampicillin/sulbactam), sulfonamides (trimethoprim and sulfamethoxazole), quinolones (ciprofloxacin and levofloxacin), and tetracycline (chloramphenicol). The disk diffusion method was conducted in accordance with the 2019 Clinical and Laboratory Standards Institute guidelines. *E*. *coli* ATCC 25922 was used as a control strain.

### Detection of ARGs and integrons

All isolates obtained from the examined samples were subjected to genotyping using polymerase chain reaction (PCR). The template DNA consisted of boiled lysates prepared from the isolates. The primer sequences, sizes of the amplified fragments, PCR conditions, and references are described in Table 1. For PCR amplification, each 25-μl reaction contained 1 μL of the DNA template, 12.5 μL of 2×Taq DNA Master Mix (CWBio, Beijing, China), 0.5 μL of each primer at a concentration of 10 μM, and 10.5 μL of ddH_2_O. PCR reactions were performed to detect the ESBL genes *bla*_CTX-M_, *bla*_CTX-M_ genotype groups 1, 2, 9, and *bla*_*TE*M_, the tetracycline resistance genes *tet(A)*, *tet(B)*, *tet(C)*, *tet(D)*, *tet(M)*, and *tet(W)*, the sulfonamide resistance genes *sul1*, *sul2*, *sul3*, and *sulA*, the chloramphenicol resistance genes *cat1*, *cmlA*, and *floR*, the colistin resistance gene *mcr-1*, and the integrase genes *intI1* (for class 1 integrons), *intI2* (for class 2 integrons), and *intI3* (for class 3 integrons). Then, the PCR products were separated by electrophoresis with a 1% agarose gel and visualized under ultraviolet light. The positive amplicons of the ARGs in most MDR strains were sequenced (Comate Bioscience Co., Ltd., Changchun, China) and the sequences were analyzed for homology using the Basic Local Alignment Search Tool (http://www.ncbi.nlm.nih.gov/BLAST/).

**Table 1:** PCR primers and conditions.

Primer	Target	Sequence (5’–3’)	Amplicon size (bp)	Source
CTX-MU1	*bla*_CTX-M_	ATGTGCAGYACCAGTAARGT	544	[24]
CTX-MU2		TGGGTRAARTARGTSACCAGA		
TEM-F	*bla*_*TEM-1*_	ATGAGTATTCAACATTTCCGT	861	[25]
TEM-R		TTACCAATGCTTAATCAGTGA		
M13-FW	*bla*_CTX-M_(CTX-M-1 group)	GGTTAAAAAATCACTGCGTC	864	[26]
M13-RV		TTGGTGACGATTTTAGCCGC		
M25-FW	*bla*_CTX-M_(CTX-M-2 group)	ATGATGACTCAGAGCATTCG	866	
M25-RV		TGGGTTACGATTTTCGCCGC		
M9-FW	*bla*_CTX-M_(CTX-M-9 group)	ATGGTGACAAAGAGAGTGCA	870	
M9-RV		CCCTTCGGCGATGATTCTC		
TetA-FW	*tet*(A)	GTAATTCTGAGCACTGTCGC	956	[27]
TetA-RV		CTGCCTGGACAACATTGCTT		
TetB-FW	*tet*(B)	CTCAGTATTCCAAGCCTTTG	414	
TetB-RV		ACTCCCCTGAGCTTGAGGGG		
TetC-FW	*tet*(C)	CTTGAGAGCCTTCAACCCAG	418	[28]
TetC-RV		ATGGTCGTCATCTACCTGCC		
TetD-FW	*tet*(D)	AAACCATTACGGCATTCTGC	787	
TetD-RV		GACCGGATACACCATCCATC		
TetM-FW	*tet*(M)	GTGGACAAAGGTACAACGAG	406	
TetM-RV		CGGTAAAGTTCGTCACACAC		
TetW-FW	*tet*(W)	GAGAGCCTGCTATATGCCAGC	168	[29]
TetW-RV		GGGCGTATCCACAATGTTAAC		
sul1-FW	*sul1*	CACCGGAAACATCGCTGCA	158	[30]
sul1-RV		AAGTTCCGCCGCAAGGCT		
sul2-FW	*sul2*	CTCCGATGGAGGCCGGTAT	190	
sul2-RV		GGGAATGCCATCTGCCTTGA		
sul3-FW	*sul3*	CCCATACCCGGATCAAGAATAA	143	
sul3-RV		CAGCGAATTGGTGCAGCTACTA		
sulA-FW	*sulA*	GCACTCCAGCAGGCTCGTAA	198	
sulA-RV		CTCTGCCACCTGACTTTTCCA		
cat1-FW	*cat1*	AACCAGACCGTTCAGCTGGAT	550	[31]
cat1-RV		CCTGCCACTCATCGCAGTAC		
cmlA-FW	*cmlA*	TGCCAGCAGTGCCGTTTAT	900	
cmlA-RV		CACCGCCCAAGCAGAAGTA		
floR-FW	*floR*	GGCTTTCGTCATTGCGTCTC	650	
floR-RV		ATCGGTAGGATGAAGGTGAGGA		
CLR5-FW	*mcr-1*	CGGTCAGTCCGTTTGTTC	400	[32]
CLR5-RV		CTTGGTCGGTCTGTAGGG		
IntI1-FW	*intI1*	ACGAGCGCAAGGTTTCGGT	565	[33]
IntI1-RV		GAAAGGTCTGGTCATACATG		
IntI2-FW	*intI2*	GTGCAACGCATTTTGCAGG	403	
IntI2-RV		CAACGGAGTCATGCAGATG		
IntI3-FW	*intI3*	CATTTGTGTTGTGGACGGC	717	
IntI3-RV		GACAGATACGTGTTTGGCAA		

Note. FW, forward; RV, reverse. Y = C or T; R = A or G.

### Statistical analyses

Statistically significant differences of isolation rate and percentage of MDR *E*. *coli* isolates among the different surveilled regions were assessed using one-way analysis of variance. All statistical analyses were conducted using IBM SPSS Statistics for Windows, version 23.0. (IBM Corporation, Armonk, NY, USA). A probability (*p*) value of < 0.01 was considered statistically significant.

## Results

### *E*. *coli* isolation

Following overnight incubation at 37°C, suspected *E*. *coli* colonies appearing with peach or reddish coloration, smooth, and wet on McConkey agar were selected for identification. The morphologic tests showed that the selected colonies were all composed of Gram-negative rod-shaped bacteria. Biochemical testing was conducted using the NMIC/ID 4 panel of the BD Phoenix™ Automated Identification and Susceptibility Testing System to obtain a more definitive identification of *E*. *coli*. Of the 1387 fecal, cloacal, and throat samples from migratory birds in six provinces in China, 478 (34.7%) *E*. *coli* isolates were obtained (Table 2). The proportions of *E*. *coli* isolates in samples collected from Zhaoqing (73.2%) and Suichuan (59.9%) were relatively high, while the proportion of *E*. *coli* isolates from Poyang Lake (17.6%) was relatively low. In terms of migratory bird species, the proportion of samples containing *E*. *coli* was higher in wading birds than swimming birds (46.2% [300/649] vs. 23.3% [149/639], respectively). With the “Aihui-Tengchong Line” as a boundary, the sampling areas were divided into northern and southern regions. The northern region included Inner Mongolia and the Ningxia Hui Autonomous Region, while all others were classified as the southern region. Although there was a significant difference in the number of samples collected between the northern and southern regions (425 vs. 962, respectively), the separation rate was similar (32.7% vs. 35.2%, respectively). The separation rate was calculated by dividing the sampling areas according to different river basins. The separation rates of the upper reaches of the Yellow River (Ningxia Hui Autonomous Region and Inner Mongolia) and the Pearl River basin (Guangdong and Guangxi provinces, respectively) were 33.1% (128/387) and 39.1% (107/274), respectively.

**Table 2:** The samples and *E*. *coli* isolates used in this study.

Source		Relationship with human habitation ^a^	Type of sample	Number of samples	Number of *E*. *coli* isolates	Isolation rate (%)
Inner Mongolia	Ordos City	B	fecal samples	212	78	36.8
	Honghaizi Wetland Park	B				
	Dali Lake	A				
Ningxia Hui Autonomous Region	Qingtongxia Nature Reserve	A	throat swabs	50	8	37.2
			cloacal swabs	50	33	
	Tianhu Wetland Park	A	throat swabs	34	8	
			cloacal swabs	34	9	
	Yellow River beach wetland	B	fecal samples	45	3	
Jiangxi	Suichuan	A	fecal samples	688	232	33.7
	Poyang Lake	A				
Guangdong	Shenzhen	B	fecal samples	204	83	40.1
	Zhaoqing	B				
	Zhanjiang	A				
Guangxi	Nanning	C	fecal samples	70	24	34.3

^a^ The relationship between sampling sites and human habitation. A: Distant; B: Close; C: Within.

### Antimicrobial resistance

In total, 118 (24.7%) of the isolates were resistant to 17 different antibiotics, while 22.6%, 16.7%, 14.4%, 13.8%, 11.1%, and 10.7% were resistant to tetracycline, ampicillin, piperacillin, trimethoprim/sulfamethoxazole, chloramphenicol, and colistin, respectively. However, fewer than 5% of the isolates were resistant to amikacin, gentamicin, cefazolin, cefotaxime, cefepime, aztreonam, amoxicillin-clavulanate, ampicillin/sulbactam, ciprofloxacin, levofloxacin, and moxifloxacin. All of the isolates were sensitive to imipenem, meropenem, ceftazidime, and piperacillin-tazobactam. Among the 118 resistant strains, 73 (61.9%) were resistant to at least three different classes of agents (Table 3). The proportion of MDR among all isolates was 15.3%. The most common MDR phenotype was tetracycline-ampicillin-piperacillin-trimethoprim/sulfamethoxazole-chloramphenicol (20.5%).

**Table 3:** Phenotypes, ARGs, and integrase genes of the *E*. *coli* isolates from migratory wild birds.

Isolate	Sampling area	Resistance patterns	Resistance genes
GN1	Nanning, Guangxi Province	TET, AMP, CHL	*tet*(A), *floR*, *cmlA*, *intl1*
GN29	Nanning, Guangxi Province	TET, AMP, CHL	*tet*(A), *floR*, *cmlA*, *intl1*
ZQ18	Zhaoqing, Guangdong Province	TET, AMP, SAN	*tet*(A), *cmlA*, *intl1*
Z30	Zhanjiang, Guangdong Province	TET, SXT, CHL	*tet*(A), *tet*(M), *sul2*, *sul3*, *floR*, *cmlA*, *intl1*
L3	Zhanjiang, Guangdong Province	TET, SXT, CHL	*tet*(A), *tet*(M), *sul3*, *cmlA*, *intl1*
GN3	Nanning, Guangxi Province	TET, CHL, GEN	*tet*(A), *tet*(B), *sul2*, *floR*, *cmlA*
ZQ13	Zhaoqing, Guangdong Province	TET, AMP, PIP, CHL	*bla*_*TEM-1*_, *tet*(A), *tet*(M), *floR*, *cmlA*, *intl1*
S11	Shenzhen, Guangdong Province	TET, AMP, PIP, CHL	*tet*(A), *floR*, *intl1*
Z31	Zhanjiang, Guangdong Province	TET, AMP, PIP, CHL	*intl1*
P41	Poyang Lake, Jiangxi Province	TET, AMP, PIP, SXT	*bla*_*TEM-1*_, *tet*(A), *sul2*, *intl1*
P42	Poyang Lake, Jiangxi Province	TET, AMP, PIP, SXT	*bla*_*TEM-1*_, *tet*(A), *sul1*, *sul2*
dachangG44	Ningxia Hui Autonomous Region	TET, AMP, PIP, SXT	*bla*_*TEM-1*_, *tet*(A),
NMJ6	Ordos City, Inner Mongolia	TET, AMP, PIP, SXT	*bla*_*TEM-1*_, *tet*(A), *sul2*, *cmlA*, *intl1*
NMJ7	Ordos City, Inner Mongolia	TET, AMP, PIP, SXT	*bla*_*TEM-1*_, *tet*(A), *sul2*, *cmlA*, *intl1*
NM15	Ordos City, Inner Mongolia	TET, AMP, PIP, SXT	*bla*_*TEM-1*_, *tet*(A), *sul2*, *intl1*
D13	Zhaoqing, Guangdong Province	TET, AMP, PIP, SXT	*bla*_*TEM-1*_, *tet*(A), *tetM*, *sul2*, *cmlA*, *intl1*
ZQ28	Zhaoqing, Guangdong Province	TET, AMP, PIP, SXT	*bla*_*TEM-1*_, *tet*(A), *sul2*, *intl1*
GN57	Nanning, Guangxi Province	TET, AMP, PIP, SXT	*bla*_*TEM-1*_, *tet*(A), *sul2*, *cmlA*, *intl1*
dc60	Suichuan, Jiangxi Province	TET, AMP, PIP, SXT	*bla*_*TEM-1*_, *tet*(A), *sul3*, *cmlA*, *intl1*
S12	Shenzhen, Guangdong Province	TET, AMP, PIP, SXT	*tet*(A), *sul2*, *intl1*
Z5	Zhanjiang, Guangdong Province	TET, AMP, PIP, SXT	*sul2*, *intl1*
Z26	Zhanjiang, Guangdong Province	TET, AMP, PIP, SXT	*tet*(A), *tetM*, *sul2*, *intl1*
P18	Poyang Lake, Jiangxi Province	TET, AMP, SAN, CHL	*tet*(A), *floR*, *cmlA*, *intl1*
NMJ1	Ordos City, Inner Mongolia	TET, AMP, SAN, CHL	*tet*(A), *floR*, *cmlA*, *intl1*
GN31	Nanning, Guangxi Province	TET, AMP, SAN, CHL	*tet*(A), *floR*, *cmlA*, *intl1*
GN67	Nanning, Guangxi Province	TET, AMP, SAN, SXT	*tet*(A), *sul2*, *cmlA*, *intl1*
ZQ15	Zhaoqing, Guangdong Province	TET, AMP, SXT, CHL	*tet*(A), *sul2*, *cmlA*, *intl1*
GN40	Nanning, Guangxi Province	TET, AMP, SXT, CHL	*tet*(A), *sul2*, *cmlA*, *intl1*
ZQ27*	Zhaoqing, Guangdong Province	TET, CFZ, AMC, SXT	*tet*(A), *sul1*, *intl1*
YO-3*	Ordos City, Inner Mongolia	AMP, CFZ, CTX, CPM, PIP	*bla*_*CTX-M*_, *bla*_*CTX-M-1*_*group*
ZQ14	Zhaoqing, Guangdong Province	TET, AMP, PIP, SXT, CHL	*bla*_*TEM-1*_, *tet*(A), *sul2*, *sul3*, *floR*, *cmlA*, *intl1*
GN24	Nanning, Guangxi Province	TET, AMP, PIP, SXT, CHL	*bla*_*TEM-1*_, *tet*(A), *sul2*, *floR*, *cmlA*, *intl1*
GN25	Nanning, Guangxi Province	TET, AMP, PIP, SXT, CHL	*bla*_*TEM-1*_, *tet*(A), *sul3*, *cmlA*, *intl1*
GN28	Nanning, Guangxi Province	TET, AMP, PIP, SXT, CHL	*bla*_*TEM-1*_, *tet*(B), *sul2*, *intl1*
GN46	Nanning, Guangxi Province	TET, AMP, PIP, SXT, CHL	*bla*_*TEM-1*_, *tet*(A), *sul2*, *floR*, *intl1*
GN64	Nanning, Guangxi Province	TET, AMP, PIP, SXT, CHL	*bla*_*TEM-1*_, *tet*(A), *sul2*, *floR*, *intl1*
dc162	Suichuan, Jiangxi Province	TET, AMP, PIP, SXT, CHL	*bla*_*TEM-1*_, *tet*(A), *sul2*, *floR*, *cmlA*, *intl1*
dc169	Suichuan, Jiangxi Province	TET, AMP, PIP, SXT, CHL	*bla*_*TEM-1*_, *tet*(A), *sul2*, *floR*, *cmlA*, *intl1*
S10	Shenzhen, Guangdong Province	TET, AMP, PIP, SXT, CHL	*tet*(A), *floR*, *intl1*
F8	Shenzhen, Guangdong Province	TET, AMP, PIP, SXT, CHL	*tet*(A), *floR*, *intl1*
F15	Shenzhen, Guangdong Province	TET, AMP, PIP, SXT, CHL	*tet*(A), *floR*, *intl1*
Z2	Zhanjiang, Guangdong Province	TET, AMP, PIP, SXT, CHL	*tet*(A), *floR*, *intl1*
Z16	Zhanjiang, Guangdong Province	TET, AMP, PIP, SXT, CHL	*tet*(A), *sul3*, *floR*, *intl1*
Z25	Zhanjiang, Guangdong Province	TET, AMP, PIP, SXT, CHL	*tet*(A), *floR*, *intl1*
L22	Zhanjiang, Guangdong Province	TET, AMP, PIP, SXT, CHL	
S34	Shenzhen, Guangdong Province	TET, AMP, SXT, CHL, SAN	*sul2*, *floR*, *cmlA*, *intl1*
dc296	Suichuan, Jiangxi Province	TET, SXT, CHL, CIP, LVX	*tet*(A), *sul2*, *floR*,
GN5	Nanning, Guangxi Province	AMP, PIP, SAN, SXT, CHL	*bla*_*TEM-1*_, *tet*(A), *tet*(M), *sul3*, *floR*, *cmlA*, *intl1*
NMB6	Ordos City, Inner Mongolia	TET, AMP, PIP, SAN, SXT, CHL	*tet*(A), *sul2*, *floR*, *cmlA*, *intl1*
GN68	Nanning, Guangxi Province	TET, AMP, PIP, SAN, SXT, CHL	*tet*(A), *floR*, *cmlA*, *intl1*
S28	Shenzhen, Guangdong Province	TET, AMP, PIP, SAN, SXT, CHL	*tet*(A), *sul2*, *floR*, *cmlA*, *intl1*
YO-5	Ordos City, Inner Mongolia	TET, AMP, PIP, SXT, CIP, LVX	*tet*(A), *sul2*, *cmlA*, *intl1*
YO-7	Ordos City, Inner Mongolia	TET, AMP, PIP, SXT, CIP, LVX	*tet*(A), *sul2*, *cmlA*, *intl1*
YO-9	Ordos City, Inner Mongolia	TET, AMP, PIP, SXT, CIP, LVX	*tet*(A), *sul2*, *cmlA*, *intl1*
YO-11	Ordos City, Inner Mongolia	TET, AMP, PIP, SXT, CIP, LVX	*tet*(A), *sul2*, *cmlA*, *intl1*
YO-55	Ordos City, Inner Mongolia	TET, AMP, PIP, SXT, CIP, LVX	*tet*(A), *sul2*, *cmlA*, *intl1*
GN23	Nanning, Guangxi Province	TET, AMP, PIP, SXT, CHL, GEN	*TEM-1*, *tet*(A), *sul3*, *cmlA*,
GN4	Nanning, Guangxi Province	TET, AMP, PIP, SXT, CHL, CIP, LVX	*bla*_*TEM-1*_, *tet*(A), *tet*(M), *sul3*, *floR*, *cmlA*, *intl1*
GN49	Nanning, Guangxi Province	TET, AMP, PIP, SXT, CHL, CIP, LVX	*bla*_*TEM-1*_, *tet*(A), *sul1*, *sul2*, *floR*, *cmlA*, *intl1*
GN65	Nanning, Guangxi Province	TET, AMP, PIP, SXT, CHL, CIP, LVX	*bla*_*TEM-1*_, *tet*(A), *sul2*, *cmlA*, *intl1*
dc70	Suichuan, Jiangxi Province	TET, AMP, PIP, SXT, CHL, CIP, LVX	*bla*_*TEM-1*_, *tet*(A), *tet*(B), *tet*(M), *sul2*, *sul3*, *floR*, *cmlA*, *intl1*
dc132	Suichuan, Jiangxi Province	TET, AMP, PIP, SXT, CHL, CIP, LVX	*bla*_*TEM-1*_, *tet*(A), *tet*(M), *sul2*, *sul3*, *floR*, *cmlA*, *intl1*
dc308	Suichuan, Jiangxi Province	TET, AMP, PIP, SXT, CHL, CIP, LVX	*bla*_*TEM-1*_, *tet*(A), *tet*(M), *sul2*, *sul3*, *floR*, *cmlA*, *intl1*
GN26	Nanning, Guangxi Province	TET, AMP, PIP, SXT, CHL, CIP, SAN	*tet*(A), *sul2*, *sul3*, *floR*, *cmlA*, *intl1*
GN11	Nanning, Guangxi Province	TET, AMP, PIP, SXT, CHL, CIP, LVX, CFZ	*bla*_*TEM-1*_, *tet*(A), *sul2*, *floR*, *cmlA*
dc114	Suichuan, Jiangxi Province	TET, AMP, PIP, SXT, CHL, CIP, LVX, CFZ, SAN	*bla*_*TEM-1*_, *tet*(A), *sul2*, *floR*, *cmlA*, *intl1*
ZQ5	Zhaoqing, Guangdong Province	TET, AMP, PIP, SAN, SXT, CHL, CIP, LVX, AMK, GEN	*bla*_*TEM-1*_, *tet*(A), *sul2*, *floR*, *cmlA*, *intl1*
ZQ22*	Zhaoqing, Guangdong Province	TET, AMP, PIP, SAN, SXT, CHL, CFZ, CTX, CPM, AZT	*bla*_*CTX-M*_, *bla*_*CTX-M-1*_*group*, *bla*_*TEM-1*_, *tet*(A), *sul2*, *floR*, *intl1*
ZQ23*	Zhaoqing, Guangdong Province	TET, AMP, PIP, SAN, SXT, CHL, CFZ, CTX, CPM, GEN	*bla*_*CTX-M*_, *bla*_*CTX-M-2*_*group*, *bla*_*CTX-M-9*_*group*, *tet*(A), *sul1*, *sul2*, *floR*, *intl1*
ZQ19*	Zhaoqing, Guangdong Province	TET, AMP, PIP, SXT, CHL, CFZ, CTX, CPM, AZT, CIP, LVX	*bla*_*CTX-M*_, *bla*_*CTX-M-1*_*group*, *bla*_*TEM-1*_, *tet*(A), *tet*(B), *sul2*, *floR*, *cmlA*, *intl1*
GN16*	Nanning, Guangxi Province	TET, AMP, PIP, SXT, CHL, CFZ, CTX, CPM, AZT, SAN, GEN	*bla*_*CTX-M*_, *bla*_*CTX-M-2*_*group*, *bla*_*CTX-M-9*_*group*, *bla*_*TEM-1*_, *tet*(A), *sul3*, *floR*, *cmlA*, *intl1*
GN27*	Nanning, Guangxi Province	TET, AMP, PIP, SXT, CHL, CFZ, CTX, CPM, AZT, SAN, GEN	*bla*_*CTX-M*_, *bla*_*CTX-M-2*_*group*, *bla*_*CTX-M-9*_*group*, *bla*_*TEM-1*_, *tet*(A), *sul3*, *floR*, *cmlA*, *intl1*
D12*	Zhaoqing, Guangdong Province	TET, AMP, PIP, SAN, SXT, CHL, CIP, LVX, GEN, CFZ, CTX, CPM, AZT	*bla*_*CTX-M*_, *bla*_*CTX-M-1*_*group*, *bla*_*CTX-M-2*_*group*, *bla*_*TEM-1*_, *tet*(A), *sul1*, *sul2*, *floR*, *cmlA*, *intl1*

Abbreviations: AMP, ampicillin; AZT, aztreonam; CHL, chloramphenicol; CIP, ciprofloxacin; CFZ, cefazolin; CPM, cefepime; CTX, cefotaxime; GEN, gentamicin; LVX, levofloxacin; PIP, piperacillin; SAN, ampicillin/ sulbactam; SXT, sulfamethoxazole/trimethoprim; TET, tetracycline.

*ESBL-producing *E*. *coli* isolates.

Among the isolates collected northwest and southeast of the Aihui-Tengchong Line, the isolation rates were uniformly distributed (32.7% and 35.2%, respectively, *p* = 0.034; Table 4), but there were significant differences in the proportions of MDR *E*. *coli* (8.6% vs. 18.0%, respectively, *p* < 0.001) and significant differences in the drug resistance rates of *E*. *coli* (21.6% vs. 26.0%, respectively, *p* = 0.006).

**Table 4:** Statistic analysis of isolation rate and drug resistance rate among *E*. *coli* isolates from different regions.

	Percentage (number of isolates/number of samples)	Percentage (number of AMR-*E*. *coli*/number of isolates)	Percentage (number of MDR-*E*. *coli*/number of isolates)
Northwest of Aihui-Tengchong Line	32.7%(139/425)	21.6%(30/139)	8.6%(12/139)
Southeast of Aihui-Tengchong Line	35.2%(339/962)	26.0%(88/339)	18.0%(61/339)
P value	0.034	0.006	< 0.001

When classified according to different river basins, the proportions of drug-resistant *E*. *coli* carried by migratory birds from highest to lowest were as follows: Pearl River (52.3%, 56/107) > Yellow River (23.4%, 30/128) > Poyang Lake (14.7%, 11/75). The resistance rates of the isolates in the Pearl River basin to tetracycline (53/57), piperacillin (48/57), ampicillin (47/57), trimethoprim-sulfamethoxazole (40/57), and chloramphenicol (40/57) were all greater than 50%. In addition, among the isolates from the Yellow River, the resistance rates of tetracycline (24/30) and ampicillin (24/16) were more than 50%, while the resistance rates of strains from the Poyang Lake basin to different antibiotics were all less than 15%. According to species traits, the drug resistance rate of *E*. *coli* from wading birds was greater than that of swimming birds (31.3% vs. 19.5%, respectively).

### ARGs and integrons

The β-lactam resistance genes *bla*_CTX-M_ and *bla*_TEM-1_, the tetracycline resistance genes *tet(*A*)*, *tet(*B*)*, and *tet(*M*)*, the sulfonamide resistance genes *sul1*, *sul2*, and *sul3*, the chloramphenicol resistance genes *cmlA* and *floR*, and the integrase gene *intI1* were identified in most of MDR isolates. Class 1 integrons were present in 72 (61.0%) of the 118 *E*. *coli* isolates, most of which were found in MDR *E*. *coli* (65/73). Homology analysis of the sequences showed that the PCR results were not false positives (S1 Table). The detection results of drug resistance phenotypes, ARGs, and integrase genes of all MDR strains are shown in Table 3.

### MIC

The E-test results showed that the highest MIC of tetracycline, ampicillin, chloramphenicol, piperacillin, amoxicillin/clavulanic acid, ampicillin/sulbactam, gentamicin, cefazolin, and amikacin was > 256 μg/ml, accounting for 5.8%, 51.2%, 35.2%, 5.8%, 0.8%, 0.8%, 6.7%, and 0.8%, respectively. The highest MIC of trimethoprim/sulfamethoxazole, ciprofloxacin, levofloxacin, and cefotaxime was > 32 μg/ml, accounting for 47.8%, 10.9%, 9.2% and 5.8%, respectively.

### ESBL-producing *E*. *coli*

Among the 73 MDR isolates, 8 (11.0%) were ESBL-producing *E*. *coli* (Table 3), which included five that carried the gene encoding TEM-1 β-lactamase (D12, ZQ19, ZQ22, GN16, and GN27). All of these isolates were resistant to both ampicillin (MIC > 256 μg/mL) and cefazolin (MIC > 256 μg/mL). Most of the CTX-M-positive *E*. *coli* isolates, with the exception of ZQ23), were resistant to ampicillin (MIC > 256 μg/mL), piperacillin (MIC > 256 μg/mL or 128 μg/mL), cefazolin (MIC > 256 μg/mL), and cefotaxime (MIC > 32 μg/mL). Notably, some ESBL-producing *E*. *coli* isolates were resistant to cefepime (MIC > 16 μg/mL), but some isolates had lower MICs (YO-3, 8 μg/mL; ZQ22, 6 μg/mL; GN16, 4 μg/mL; ZQ23, 2 μg/mL; GN27, 2 μg/mL). Most of the ESBL-producing *E*. *coli* isolates (7/8) were from samples collected from the Pearl River Basin. In regard to the classification of migratory birds, most hosts of the ESBL-producing isolates (6/8) were members of the order *Ciconiiformes* (ZQ19, ZQ22, ZQ23, ZQ27, GN16, and GN27), and the rest were members of the orders *Gruiformes* (D12) and *Charadriiformes* (YO-3).

## Discussion

*E*. *coli* is an important pathogen that causes severe infections in humans and animals, and acts as a donor and as a recipient of AGRs involving other bacteria. The main mechanisms of AMR among *E*. *coli* strains include (a) inactivation of antibiotics by producing inactivating enzymes or hydrolases; (b) changes to antibiotic target sites; (c) changes to bacterial membrane permeability; and (d) resistance associated with drug efflux pumps. *E*. *coli* has a great capacity to accumulate ARGs, mostly through horizontal gene transfer. Some mobile genetic elements seem to play a major role in the dissemination of ARGs. In general, antimicrobial resistance in *E*. *coli* is considered a major challenges in both humans and animals and must be considered as an urgent public health concern [34].

Many studies have shown that migratory birds transport antibiotic-resistant bacteria over long distances [3,35]. The East-Asian Australasian flyway is considered to be used by the most species of migratory birds [19]. Migratory birds can acquire and transmit MDR bacteria along the long migratory journey from Siberia to Australia [36]. A study conducted in Russia detected high levels of resistance to critically important antimicrobials, such as extended-spectrum cephalosporins, fluoroquinolones, colistin, and carbapenems, in wild birds [37]. The sampling sites in this study were located along this migratory route and were divided into two geographic locations by the Aihui-Tengchong Line. Almost half (43.8%) of the land southeast of the Aihui Tengchong line is inhabited by 94.1% of the population in China. The Aihui Tengchong line has also become the dividing line of urbanization level of China to some extent. In this study, the distribution of MDR *E*. *coli* was greater southeast of the Aihui-Tengchong Line than northwest. The significant difference in the drug resistance rate among the *E*. *coli* isolates collected from northern and southern China might be related to the impact of various human activities.

The significant difference in the drug resistance rate among *E*. *coli* isolates between swimming and wading birds is likely related to the difference in environments and feeding habits of migratory birds. Various birds previously identified as carriers of ESBL-producing *E*. *coli* are considerably mobile and often cross continents [38,39]. Among the eight ESBL-positive isolates, seven were from the Pearl River basin, and all were collected from wading birds. Wading birds mainly feed underwater or on underwater sediments, such as sludge, which may be related to the presence of drug resistance genes. To date, relatively few studies have investigated MDR bacteria carried by wading birds. Thus, follow-up analysis based on these results is warranted.

Overall, the prevalence of MDR *E*. *coli* was higher in the eastern and southern sampling sites than in the northwest, which was also consistent with the antibiotic emission density in China [34]. The level of drug resistance in a certain area is closely related to specific regional factors, such as local economic and agricultural development. Samples collected from the Pearl River basin had the highest level of drug resistance possibly because of the high discharge of antibiotics and industrial sewage in the region [40,41]. Notably, the high levels of antibiotics in most of the sampling sites in the Pearl River basin were due to closer proximity to human habitats or by birds feeding on human garbage [37]. Previous studies have shown that the concentrations of quinolones, macrolides, and β-lactams are much higher in the sediments of the Pearl River basin as compared to those of the Yellow River and Yangtze River basins [42,43].

Strains isolated from the Yellow River basin were most commonly resistant to tetracycline, followed by β-lactams. These results are basically consistent with those of previous studies on the content of antibiotics in drinking water in the Yellow River basin and coastal cities [42,44]. Tetracyclines were the first major category of broad-spectrum antibiotics used in humans and animals globally [45]. In general, *E*. *coli* of animal origin are often resistant to older antimicrobial agents, including tetracyclines and sulfonamides. The active efflux gene tetA and ribosomal protection gene tetM detected in this study can be transferred between bacteria through plasmids and transposons, resulting in extensive drug resistance [46]. Among all the sampling sites in this study, no drug-resistant *E*. *coli* was isolated from the Dali Lake samples, which was likely due to the distances of the sampling sites from human habitats, as these areas had lower concentrations of antibiotics in the environment and, thus, little impact on migratory birds.

The samples from the Yellow River basin not only contained more tet(A) genes, but also a certain amount of intI1. Integrons can rapidly obtain and disseminate various genes encoding resistance to antibiotics [47,48] and are classified as class 1, 2, or 3 based on the integrase gene (intI). Class 1 integrons are the most common and, thus, were monitored in this study. Interestingly, intI1 was detected in 88.7% (63/71) of MDR *E*. *coli* in the present study, which seems to support the idea that the occurrence of multidrug resistance among microbes is associated with mobile genetic elements [49].

The wetland area of Poyang Lake is among the top 10 ecological conservation areas in China and also the largest bird reserve and habitat for migratory birds in the world [50]. The isolation rate of drug-resistant bacteria from samples collected from birds around Poyang Lake was low (14.7%), which may reflect the low antibiotic emission in this area. A previous study reported that the concentrations of antibiotics around Poyang Lake are relatively moderate to below average as compared to other lakes in China [51]. Although the prevalence of drug-resistant bacteria around Poyang Lake area was low, considering the high mobility of migratory birds and the important geographical location of Poyang Lake, the levels of antibiotics in this area should be closely monitored. The dominant genes in the Poyang Lake samples were the tetracycline resistance gene *tet*(A), ESBL gene *bla*_*TEM-1*_, and sulfonamide resistance gene *sul2*, which is generally consistent with the findings of previous studies [52]. Sulfonamide, tetracycline, and quinolone resistance genes are the most frequently detected ARGs in lakes and rivers and, therefore, have been suggested as possible indicators of environment pollution of antibiotics [53]. In addition, tetracyclines and sulfonamides (i.e., sulfadiazine, sulfamethoxazole, sulfamethazine, and sulfachlorpyridazine) are considered as priorities for control of antibiotics [54]. However, although migratory birds in different areas were sampled, this study did not take into consideration the timing in the same environment.

## Conclusion

The result of this study confirmed the relationships of migratory birds with the environment and the spread of bacterial drug resistance. Migratory wild birds carrying MDR *E*. *coli* might be act as potential transmitters of antimicrobial resistance in China. Whether the drug-resistant bacteria carried by these migratory birds can colonize the host for long periods and spread with migration remains to be further studied. The results also demonstrated regional differences in MDR *E*. *coli* carried by migratory birds in China and the drug resistance rate was closely related to the population density and antibiotic emission density of different drainage areas. Although migratory birds, as carriers of drug-resistant bacteria, have a limited influence on the environment, the long-term impact should not be ignored. Recent works have shown that even treated waste can impact the acquisition of ARGs by avian wildlife [36]. Therefore, it is not only necessary to pay attention to the important role of migratory birds in the transmission of drug-resistant bacteria, but also to reduce the use of antibiotics in order to fundamentally reduce the transmission of ARGs.

## Supporting information

S1 Table The sequences of the ARGs of the MDR *E*. *coli* strains. (DOCX)
