# Supplementary material for: Migratory wild birds carrying multidrug-resistant Escherichia coli as potential transmitters of antimicrobial resistance in China
Source: PLoS One. 2021 Dec 15;16(12):e0261444. doi: 10.1371/journal.pone.0261444 (PMC8673662; doi:10.1371/journal.pone.0261444)
Supplement: S1 Table — (DOCX) [file pone.0261444.s001.docx]

**S1 Table. The sequences of the antimicrobial resistance genes in multidrug resistant strains**

| **Resistance gene** | **Isolate** | **Sequence** | **Reference sequence**  **(accession number)** | **Homology**  **(%)** |
| --- | --- | --- | --- | --- |
| *bla*_CTX-M_ | D12 | TGGGTGAAGTAAGTGACCAGAATCAGCGGCGCACGATCTTTTGGCCAGATCACCGCGATATCGTTGGTGGTGCCATAGCCACCGCTGCCGGTTTTATCCCCCACAACCCAGGAAGCAGGCAGTCCAGCCCGAATGCTGGCTGCGCCGGTCGTATTGCCTTTGAGCCACGTCACCAACTGCGCCCGCTGGGTTTCGCCCAGCGCATGACCCAGCGTAAGCTGACGCAACGTCTGCGCCATCGCCCGCGGCGTGGTGGTGTCTCTCGGGTCGCCGGGAATGGCGGTATTCAGCGTAGGTTCAGTGCGATCCAGACGAAACGTCTCATCGCCGATCGCGCGGGCAAAAGCCGTCACGCCTCCCGGGCCACCGAGCTGGGCAATCAATTTGTTCATGGCGGTATTGTCGCTGTACTGCAACGCGGCCGCGCTCAGTTCTGCCAGCGTCATTGTGCCGTTGACGTGTTTTTCGGCAATCGGATTGTAGTTAACCAGATCGGCAGGCTTGATCTCGACAGGCTGATTAAGCAGCTGCTTTTGCGTTTCACTCTGCTTAAGCACCGCCGCGGCCGCCATAACTTTACTGGTACTGCACAT | [MK704494](https://www.ncbi.nlm.nih.gov/nuccore/MK704494) | 100.00 |
|  | ZQ19 | TGAAGTAAGTGACCAGAATCAGCGGCGCACGATCTTTTGGCCAGATCACCGCGATATCGTTGGTGGTGCCATAGCCACCGCTGCCGGTTTTATCCCCCACAACCCAGGAAGCAGGCAGTCCAGCCTGAATGCTCGCTGCACCGGTGGTATTGCCTTTCATCCATGTCACCAGCTGCGCCCGTTGGCTGTCGCCCAATGCTTTACCCAGCGTCAGATTCCGCAGAGTTTGCGCCATTGCCCGAGGTGAAGTGGTATCACGCGGATCGCCCGGAATGGCGGTGTTTAACGTCGGCTCGGTACGGTCGAGACGGAACGTTTCGTCTCCCAGCTGTCGGGCGAACGCGGTGACGCTAGCCGGGCCGCCAACGTGAGCAATCAGCTTATTCATCGCCACGTTATCGCTGTACTGTAGCGCGGCCGCGCTAAGCTCAGCCAGTGACATCGTCCCATTGACGTGCTTTTCCGCAATCGGATTATAGTTAACAAGGTCAGATTTTTTGATCTCAACTCGCTGATTTAACAGATTCGGTTCGCTTTCACTTTTCTTCAGCACCGCGGCCACGGCCATCACTTTACTGGTGCTGCACAT | [AP023198](https://www.ncbi.nlm.nih.gov/nuccore/AP023198) | 100.00 |
|  | ZQ22 | GGTGAAGTAAGTGACCAGAATCAGCGGCGCACGATCTTTTGGCCAGATCACCGCGATATCGTTGGTGGTGCCATAGCCACCGCTGCCGGTTTTATCCCCCACAACCCAGGAAGCAGGCAGTCCAGCCTGAATGCTCGCTGCACCGGTGGTATTGCCTTTCATCCATGTCACCAGCTGCGCCCGTTGGCTGTCGCCCAATGCTTTACCCAGCGTCAGATTCCGCAGAGTTTGCGCCATTGCCCGAGGTGAAGTGGTATCACGCGGATCGCCCGGAATGGCGGTGTTTAACGTCGGCTCGGTACGGTCGAGACGGAACGTTTCGTCTCCCAGCTGTCGGGCGAACGCGGTGACGCTAGCCGGGCCGCCAACGTGAGCAATCAGCTTATTCATCGCCACGTTATCGCTGTACTGTAGCGCGGCCGCGCTAAGCTCAGCCAGTGACATCGTCCCATTGACGTGCTTTTCCGCAATCGGATTATAGTTAACAAGGTCAGATTTTTTGATCTCAACTCGCTGATTTAACAGATTCGGTTCGCTTTCACTTTTCTTCAGCACCGCGGCCACGGCCATCACTTTACTGGTGCTGCACAT | [AP023198](https://www.ncbi.nlm.nih.gov/nuccore/AP023198) | 100.00 |
|  | GN16 | GCGGCCAGATCACCGCAATATCATTGGTGGTGCCGTAGTCGCCGCTGCCGGTCTTATCACCCACAGTCCACGACGTCGGTAAGCCGGCCCGAATGCTGGCTGCGCCGGTCGTATTGCCTTTGAGCCACGTCACCAACTGCGCCCGCTGGGTTTCGCCCAGCGCATGACCCAGCGTAAGCTGACGCAACGTCTGCGCCATCGCCCGCGGCGTGGTGGTGTCTCTCGGGTCGCCGGGAATGGCGGTATTCAGCGTAGGTTCAGTGCGATCCAGACGAAACGTCTCATCGCCGATCGCGCGGGCAAAAGCCGTCACGCCTCCCGGGCCACCGAGCTGGGCAATCAATTTGTTCATGGCGGTATTGTCGCTGTACTGCAACGCGGCCGCGCTCAGTTCTGCCAGCGTCATTGTGCCGTTGACGTGTTTTTCGGCAATCGGATTGTAGTTAACCAGATCGGCAGGCTTGATCTCGACAGGCTGATTAAGCAGCTGCTTTTGCGTTTCACTCTGCTTAAGCACCGCCGCGACCGCCATAACTTTACTGGTACTGCACAT | [CP083417](https://www.ncbi.nlm.nih.gov/nuccore/CP083417) | 100.00 |
|  | GN27 | GCGCACGACCCTGCGGCCAGATCACCGCAATATCATTGGTGGTGCCGTAGTCGCCGCTGCCGGTCTTATCACCCACAGTCCACGACGTCGGTAAGCCGGCCCGAATGCTGGCTGCGCCGGTCGTATTGCCTTTGAGCCACGTCACCAACTGCGCCCGCTGGGTTTCGCCCAGCGCATGACCCAGCGTAAGCTGACGCAACGTCTGCGCCATCGCCCGCGGCGTGGTGGTGTCTCTCGGGTCGCCGGGAATGGCGGTATTCAGCGTAGGTTCAGTGCGATCCAGACGAAACGTCTCATCGCCGATCGCGCGGGCAAAAGCCGTCACGCCTCCCGGGCCACCGAGCTGGGCAATCAATTTGTTCATGGCGGTATTGTCGCTGTACTGCAACGCGGCCGCGCTCAGTTCTGCCAGCGTCATTGTGCCGTTGACGTGTTTTTCGGCAATCGGATTGTAGTTAACCAGATCGGCAGGCTTGATCTCGACAGGCTGATTAAGCAGCTGCTTTTGCGTTTCACTCTGCTTAAGCACCGCCGCGACCGCCATAACTTTACTGGTACTGCACAT | [CP083417](https://www.ncbi.nlm.nih.gov/nuccore/CP083417) | 100.00 |
| *bla_TEM-1_* | NMB6 | TTACCAATGCTTAATCAGTGAGGCACCTATCTCAGCGATCTGTCTATTTCGTTCATCCATAGTTGCCTGACTCCCCGTCGTGTAGATAACTACGATACGGGAGGGCTTACCATCTGGCCCCAGTGCTGCAATGATACCGCGAGACCCACGCTCACCGGCTCCAGATTTATCAGCAATAAACCAGCCAGCCGGAAGGGCCGAGCGCAGAAGTGGTCCTGCAACTTTATCCGCCTCCATCCAGTCTATTAATTGTTGCCGGGAAGCTAGAGTAAGTAGTTCGCCAGTTAATAGTTTGCGCAACGTTGTTGCCATTGCTGCAGGCATCGTGGTGTCACGCTCGTCGTTTGGTATGGCTTCATTCAGCTCCGGTTCCCAACGATCAAGGCGAGTTACATGATCCCCCATGTTGTGCAAAAAAGCGGTTAGCTCCTTCGGTCCTCCGATCGTTGTCAGAAGTAAGTTGGCCGCAGTGTTATCACTCATGGTTATGGCAGCACTGCATAATTCTCTTACTGTCATGCCATCCGTAAGATGCTTTTCTGTGACTGGTGAGTACTCAACCAAGTCATTCTGAGAATAGTGTATGCGGCGACCGAGTTGCTCTTGCCCGGCGTCAACACGGGATAATACCGCGCCACATAGCAGAACTTTAAAAGTGCTCATCATTGGAAAACGTTCTTCGGGGCGAAAACTCTCAAGGATCTTACCGCTGTTGAGATCCAGTTCGATGTAACCCACTCGTGCACCCAACTGATCTTCAGCATCTTTTACTTTCACCAGCGTTTCTGGGTGAGCAAAAACAGGAAGGCAAAATGCCGCAAAAAAGGGAATAAGGGCGACACGGAAATGTTGAATACTCAT | [CP055262](https://www.ncbi.nlm.nih.gov/nuccore/CP055262) | 100.00 |
|  | ZQ19 | AGGAAGAGTATGAGTATTCAACATTTTCGTGTCGCCCTTATTCCCTTTTTTGCGGCATTTTGCCTTCCTGTTTTTGCTCACCCAGAAACGCTGGTGAAAGTAAAAGATGCTGAAGATCAGTTGGGTGCACGAGTGGGTTACATCGAACTGGATCTCAACAGCGGTAAGATCCTTGAGAGTTTTCGCCCCGAAGAACGTTTTCCAATGATGAGCACTTTTAAAGTTCTGCTATGTGGTGCGGTATTATCCCGTGTTGACGCCGGGCAAGAGCAACTCGGTCGCCGCATACACTATTCTCAGAATGACTTGGTTGAGTACTCACCAGTCACAGAAAAGCATCTTACGGATGGCATGACAGTAAGAGAATTATGCAGTGCTGCCATAACCATGAGTGATAACACTGCTGCCAACTTACTTCTGACAACGATCGGAGGACCGAAGGAGCTAACCGCTTTTTTGCACAACATGGGGGATCATGTAACTCGCCTTGATCGTTGGGAACCGGAGCTGAATGAAGCCATACCAAACGACGAGCGTGACACCACGATGCCTGCAGCAATGGCAACAACGTTGCGCAAACTATTAACTGGCGAACTACTTACTCTAGCTTCCCGGCAACAATTAATAGACTGGATGGAGGCGGATAAAGTTGCAGGACCACTTCTGCGCTCGGCCCTTCCGGCTGGCTGGTTTATTGCTGATAAATCTGGAGCCGGTGAGCGTGGGTCTCGCGGTATCATTGCAGCACTGGGGCCAGATGGTAAGCCCTCCCGTATCGTAGTTATCTACACGACGGGGAGTCAGGCAACTATGGATGAACGAAATAGACAGATCGCTGAGATAGGTGCCTCACTGATTAAGCATTGGTAA | [CP066845](https://www.ncbi.nlm.nih.gov/nuccore/CP066845) | 100.00 |
|  | dc308 | ATTCAACATTTTCGTGTCGCCCTTATTCCCTTTTTTGCGGCATTTTGCCTTCCTGTTTTTGCTCACCCAGAAACGCTGGTGAAAGTAAAAGATGCTGAAGATCAGTTGGGTGCACGAGTGGGTTACATCGAACTGGATCTCAACAGCGGTAAGATCCTTGAGAGTTTTCGCCCCGAAGAACGTTTTCCAATGATGAGCACTTTTAAAGTTCTGCTATGTGGTGCGGTATTATCCCGTGTTGACGCCGGGCAAGAGCAACTCGGTCGCCGCATACACTATTCTCAGAATGACTTGGTTGAGTACTCACCAGTCACAGAAAAGCATCTTACGGATGGCATGACAGTAAGAGAATTATGCAGTGCTGCCATAACCATGAGTGATAACACTGCTGCCAACTTACTTCTGACAACGATCGGAGGACCGAAGGAGCTAACCGCTTTTTTGCACAACATGGGGGATCATGTAACTCGCCTTGATCGTTGGGAACCGGAGCTGAATGAAGCCATACCAAACGACGAGCGTGACACCACGATGCCTGCAGCAATGGCAACAACGTTGCGCAAACTATTAACTGGCGAACTACTTACTCTAGCTTCCCGGCAACAATTAATAGACTGGATGGAGGCGGATAAAGTTGCAGGACCACTTCTGCGCTCGGCCCTTCCGGCTGGCTGGTTTATTGCTGATAAATCTGGAGCCGGTGAGCGTGGGTCTCGCGGTATCATTGCAGCACTGGGGCCAGATGGTAAGCCCTCCCGTATCGTAGTTATCTACACGACGGGGAGTCAGGCAACTATGGATGAACGAAATAGACAGATCGCTGAGATAGGTGCCTCACTGATTAAGCATTGGTAA | [CP066845](https://www.ncbi.nlm.nih.gov/nuccore/CP066845) | 100.00 |
|  | GN4 | AAAGGAAGAGTATGAGTATTCAACATTTTCGTGTCGCCCTTATTCCCTTTTTTGCGGCATTTTGCCTTCCTGTTTTTGCTCACCCAGAAACGCTGGTGAAAGTAAAAGATGCTGAAGATCAGTTGGGTGCACGAGTGGGTTACATCGAACTGGATCTCAACAGCGGTAAGATCCTTGAGAGTTTTCGCCCCGAAGAACGTTTTCCAATGATGAGCACTTTTAAAGTTCTGCTATGTGGTGCGGTATTATCCCGTGTTGACGCCGGGCAAGAGCAACTCGGTCGCCGCATACACTATTCTCAGAATGACTTGGTTGAGTACTCACCAGTCACAGAAAAGCATCTTACGGATGGCATGACAGTAAGAGAATTATGCAGTGCTGCCATAACCATGAGTGATAACACTGCTGCCAACTTACTTCTGACAACGATCGGAGGACCGAAGGAGCTAACCGCTTTTTTGCACAACATGGGGGATCATGTAACTCGCCTTGATCGTTGGGAACCGGAGCTGAATGAAGCCATACCAAACGACGAGCGTGACACCACGATGCCTGCAGCAATGGCAACAACGTTGCGCAAACTATTAACTGGCGAACTACTTACTCTAGCTTCCCGGCAACAATTAATAGACTGGATGGAGGCGGATAAAGTTGCAGGACCACTTCTGCGCTCGGCCCTTCCGGCTGGCTGGTTTATTGCTGATAAATCTGGAGCCGGTGAGCGTGGGTCTCGCGGTATCATTGCAGCACTGGGGCCAGATGGTAAGCCCTCCCGTATCGTAGTTATCTACACGACGGGGAGTCAGGCAACTATGGATGAACGAAATAGACAGATCGCTGAGATAGGTGCCTCACTGATTAAGCATTGGTAA | [CP066845](https://www.ncbi.nlm.nih.gov/nuccore/CP066845) | 100.00 |
|  | P42 | GTATGAGTATTCAACATTTTCGTGTCGCCCTTATTCCCTTTTTTGCGGCATTTTGCCTTCCTGTTTTTGCTCACCCAGAAACGCTGGTGAAAGTAAAAGATGCTGAAGATCAGTTGGGTGCACGAGTGGGTTACATCGAACTGGATCTCAACAGCGGTAAGATCCTTGAGAGTTTTCGCCCCGAAGAACGTTTTCCAATGATGAGCACTTTTAAAGTTCTGCTATGTGGTGCGGTATTATCCCGTGTTGACGCCGGGCAAGAGCAACTCGGTCGCCGCATACACTATTCTCAGAATGACTTGGTTGAGTACTCACCAGTCACAGAAAAGCATCTTACGGATGGCATGACAGTAAGAGAATTATGCAGTGCTGCCATAACCATGAGTGATAACACTGCTGCCAACTTACTTCTGACAACGATCGGAGGACCGAAGGAGCTAACCGCTTTTTTGCACAACATGGGGGATCATGTAACTCGCCTTGATCGTTGGGAACCGGAGCTGAATGAAGCCATACCAAACGACGAGCGTGACACCACGATGCCTGCAGCAATGGCAACAACGTTGCGCAAACTATTAACTGGCGAACTACTTACTCTAGCTTCCCGGCAACAATTAATAGACTGGATGGAGGCGGATAAAGTTGCAGGACCACTTCTGCGCTCGGCCCTTCCGGCTGGCTGGTTTATTGCTGATAAATCTGGAGCCGGTGAGCGTGGGTCTCGCGGTATCATTGCAGCACTGGGGCCAGATGGTAAGCCCTCCCGTATCGTAGTTATCTACACGACGGGGAGTCAGGCAACTATGGATGAACGAAATAGACAGATCGCTGAGATAGGTGCCTCACTGATTAAGCATTGGTAA | [CP066845](https://www.ncbi.nlm.nih.gov/nuccore/CP066845) | 100.00 |
| *tet*(A) | P42 | GTGCTGTTCGCTCGGCTGATTATGCCGGTGCTGCCGGGCCTCCTGCGCGATCTGGTTCACTCGAACGACGTCACCGCCCACTATGGCATTCTGCTGGCGCTGTATGCGTTGATGCAATTTGCCTGCGCACCTGTGCTGGGCGCGCTGTCGGATCGTTTCGGGCGGCGGCCGGTCTTGCTCGTCTCGCTGGCCGGCGCTGCTGTCGACTACGCCATCATGGCGACGGCGCCTTTCCTTTGGGTTCTCTATATCGGGCGGATCGTGGCCGGCATCACCGGGGCGACTGGGGCGGTAGCCGGCGCTTATATTGCCGATATCACTGATGGCGATGAGCGCGCGCGGCACTTCGGCTTCATGAGCGCCTGTTTCGGGTTCGGGATGGTCGCGGGACCTGTGCTCGGTGGGCTGATGGGCGGTTTCTCCCCCCACGCTCCGTTCTTCGCCGCGGCAGCCTTGAACGGCCTCAATTTCCTGACGGGCTGTTTCCTTTTGCCGGAGTCGCACAAAGGCGAACGCCGGCCGTTACGCCGGGAGGCTCTCAACCCGCTCGCTTCGTTCCGGTGGGCCCGGGGCATGACCGTCGTCGCCGCCCTGATGGCGGTCTTCTTCATCATGCAACTTGTCGGACAGGTGCCGGCCGCGCTTTGGGTCATTTTCGGCGAGGATCGCTTTCACTGGGACGCGACCACGATCGGCATTTCGCTTGCCGCATTTGGCATTCTGCATTCACTCGCCCAGGCAATGATCACCGGCCCTGTAGCCGCCCGGCTCGGCGAAAGGCGGGCACTCATGCTCGGAATGATTGCCGACGGCACAGGCTACATCCTGCTTGCCTTCGCGACACGGGGATGGATGGCGTTCCCGATCATGGTCCTGCTTGCTTCGGGTGGCATCGGAATGCCGGCGCTGCAGCATGGT | [MW574938](https://www.ncbi.nlm.nih.gov/nuccore/MW574938) | 100.00 |
|  | P104 | GTGGCGGTTCGCTCGGCTGATTATGCCGGTGCTGCCGGGCCTCCTGCGCGATCTGGTTCACTCGAACGACGTCGCCGCCCACTATGGCATTCTGCTGGCGCTGTATGCGTTGATGCAATTTGCCTGCGCACCTGTGCTGGGCGCGCTGTCGGATCGTTTCGGGCGGCGGCCGGTCTTGCTCGTCTCGCTGGCCGGCGCTGCTGTCGACTACGCCATCATGGCGACGGCGCCTTTCCTTTGGGTTCTCTATATCGGGCGGATCGTGGCCGGCATCACCGGGGCGACTGGGGCGGTAGCCGGCGCTTATATTGCCGATATCACTGATGGCGATGAGCGCGCGCGGCACTTCGGCTTCATGAGCGCCTGTTTCGGGTTCGGGATGGTCGCGGGACCTGTGCTCGGTGGGCTGATGGGCGGTTTCTCCCCCCACGCTCCGTTCTTCGCCGCGGCAGCCTTGAACGGCCTCAATTTCCTGACGGGCTGTTTCCTTTTGCCGGAGTCGCACAAAGGCGAACGCCGGCCGTTACGCCGGGAGGCTCTCAACCCGCTCGCTTCGTTCCGGTGGGCCCGGGGCATGACCGTCGTCGCCGCCCTGATGGCGGTCTTCTTCATCATGCAACTTGTCGGACAGGTGCCGGCCGCGCTTTGGGTCATTTTCGGCGAGGATCGCTTTCACTGGGACGCGACCACGATCGGCATTTCGCTTGCCGCATTTGGCATTCTGCATTCACTCGCCCAGGCAATGATCACCGGCCCTGTAGCCGCCCGGCTCGGCGAAAGGCGGGCACTCATGCTCGGAATGATTGCCGACGGCACAGGCTACATCCTGCTTGCCTTCGCGACACGGGGATGGATGGCGTTCCCGATCATGGTCCTGCTTGCTTCGGGTGGCATCGGAATGCCGGCGCTGCAGATGTGT | [CP055262](https://www.ncbi.nlm.nih.gov/nuccore/CP055262) | 99.89 |
|  | NMJ1 | CGCATTCGGCTCGGCCTGATTATGCCGGTGCTGCCGGGCCTCCTGCGCGATCTGGTTCACTCGAACGACGTCACCGCCCACTATGGCATTCTGCTGGCGCTGTATGCGTTGATGCAATTTGCCTGCGCACCTGTGCTGGGCGCGCTGTCGGATCGTTTCGGGCGGCGGCCGGTCTTGCTCGTCTCGCTGGCCGGCGCTGCTGTCGACTACGCCATCATGGCGACGGCGCCTTTCCTTTGGGTTCTCTATATCGGGCGGATCGTGGCCGGCATCACCGGGGCGACTGGGGCGGTAGCCGGCGCTTATATTGCCGATATCACTGATGGCGATGAGCGCGCGCGGCACTTCGGCTTCATGAGCGCCTGTTTCGGGTTCGGGATGGTCGCGGGACCTGTGCTCGGTGGGCTGATGGGCGGTTTCTCCCCCCACGCTCCGTTCTTCGCCGCGGCAGCCTTGAACGGCCTCAATTTCCTGACGGGCTGTTTCCTTTTGCCGGAGTCGCACAAAGGCGAACGCCGGCCGTTACGCCGGGAGGCTCTCAACCCGCTCGCTTCGTTCCGGTGGGCCCGGGGCATGACCGTCGTCGCCGCCCTGATGGCGGTCTTCTTCATCATGCAACTTGTCGGACAGGTGCCGGCCGCGCTTTGGGTCATTTTCGGCGAGGATCGCTTTCACTGGGACGCGACCACGATCGGCATTTCGCTTGCCGCATTTGGCATTCTGCATTCACTCGCCCAGGCAATGATCACCGGCCCTGTAGCCGCCCGGCTCGGCGAAAGGCGGGCACTCATGCTCGGAATGATTGCCGACGGCACAGGCTACATCCTGCTTGCCTTCGCGACACGGGGATGGATGGCGTTCCCGATCATGGTCCTGCTTGCTTCGGGTGGCATCGGAATGCCGGCGCTGCAGATGT | [MZ475699](https://www.ncbi.nlm.nih.gov/nuccore/MZ475699) | 99.89 |
|  | NMJ2 | GGCAGTTTGCTCGGCTGATTATGCCGGTGCTGCCGGGCCTCCTGCGCGATCTGGTTCACTCGAACGACGTCACCGCCCACTATGGCATTCTGCTGGCGCTGTATGCGTTGATGCAATTTGCCTGCGCACCTGTGCTGGGCGCGCTGTCGGATCGTTTCGGGCGGCGGCCGGTCTTGCTCGTCTCGCTGGCCGGCGCTGCTGTCGACTACGCCATCATGGCGACGGCGCCTTTCCTTTGGGTTCTCTATATCGGGCGGATCGTGGCCGGCATCACCGGGGCGACTGGGGCGGTAGCCGGCGCTTATATTGCCGATATCACTGATGGCGATGAGCGCGCGCGGCACTTCGGCTTCATGAGCGCCTGTTTCGGGTTCGGGATGGTCGCGGGACCTGTGCTCGGTGGGCTGATGGGCGGTTTCTCCCCCCACGCTCCGTTCTTCGCCGCGGCAGCCTTGAACGGCCTCAATTTCCTGACGGGCTGTTTCCTTTTGCCGGAGTCGCACAAAGGCGAACGCCGGCCGTTACGCCGGGAGGCTCTCAACCCGCTCGCTTCGTTCCGGTGGGCCCGGGGCATGACCGTCGTCGCCGCCCTGATGGCGGTCTTCTTCATCATGCAACTTGTCGGACAGGTGCCGGCCGCGCTTTGGGTCATTTTCGGCGAGGATCGCTTTCACTGGGACGCGACCACGATCGGCATTTCGCTTGCCGCATTTGGCATTCTGCATTCACTCGCCCAGGCAATGATCACCGGCCCTGTAGCCGCCCGGCTCGGCGAAAGGCGGGCACTCATGCTCGGAATGATTGCCGACGGCACAGGCTACATCCTGCTTGCCTTCGCGACACGGGGATGGATGGCGTTCCCGATCATGGTCCTGCTTGCTTCGGGTGGCATCGGAATGCCGGCGCTGCAAGCATG | [MZ475699](https://www.ncbi.nlm.nih.gov/nuccore/MZ475699) | 99.89 |
|  | NMJ3 | CGGCGGTTCGCTCGGCTGATTATGCCGGTGCTGCCGGGCCTCCTGCGCGATCTGGTTCACTCGAACGACGTCACCGCCCACTATGGCATTCTGCTGGCGCTGTATGCGTTGATGCAATTTGCCTGCGCACCTGTGCTGGGCGCGCTGTCGGATCGTTTCGGGCGGCGGCCGGTCTTGCTCGTCTCGCTGGCCGGCGCTGCTGTCGACTACGCCATCATGGCGACGGCGCCTTTCCTTTGGGTTCTCTATATCGGGCGGATCGTGGCCGGCATCACCGGGGCGACTGGGGCGGTAGCCGGCGCTTATATTGCCGATATCACTGATGGCGATGAGCGCGCGCGGCACTTCGGCTTCATGAGCGCCTGTTTCGGGTTCGGGATGGTCGCGGGACCTGTGCTCGGTGGGCTGATGGGCGGTTTCTCCCCCCACGCTCCGTTCTTCGCCGCGGCAGCCTTGAACGGCCTCAATTTCCTGACGGGCTGTTTCCTTTTGCCGGAGTCGCACAAAGGCGAACGCCGGCCGTTACGCCGGGAGGCTCTCAACCCGCTCGCTTCGTTCCGGTGGGCCCGGGGCATGACCGTCGTCGCCGCCCTGATGGCGGTCTTCTTCATCATGCAACTTGTCGGACAGGTGCCGGCCGCGCTTTGGGTCATTTTCGGCGAGGATCGCTTTCACTGGGACGCGACCACGATCGGCATTTCGCTTGCCGCATTTGGCATTCTGCATTCACTCGCCCAGGCAATGATCACCGGCCCTGTAGCCGCCCGGCTCGGCGAaAGGCGGGCACTCATGCTCGGAATGATTGCCGACGGCACAGGCTACATCCTGCTTGCCTTCGCGACACGGGGATGGATGGCGTTCCCGATCATGGTCCTGCTTGCTTCGGGTGGCATCGGAATGCCGGCGCTGCAAGCAT | [MZ475699](https://www.ncbi.nlm.nih.gov/nuccore/MZ475699) | 99.89 |
|  | NMJ6 | GTGCAAGTTCGCTCGGCTGATTATGCCGGTGCTGCCGGGCCTCCTGCGCGATCTGGTTCACTCGAACGACGTCACCGCCCACTATGGCATTCTGCTGGCGCTGTATGCGTTGATGCAATTTGCCTGCGCACCTGTGCTGGGCGCGCTGTCGGATCGTTTCGGGCGGCGGCCGGTCTTGCTCGTCTCGCTGGCCGGCGCTGCTGTCGACTACGCCATCATGGCGACGACGCCTTTCCTTTGGGTTCTCTATATCGGGCGGATCGTGGCCGGCATCACCGGGGCGACTGGGGCGGTAGCCGGCGCTTATATTGCCGATATCACTGATGGCGATGAGCGCGCGCGGCACTTCGGCTTCATGAGCGCCTGTTTCGGGTTCGGGATGGTCGCGGGACCTGTGCTCGGTGGGCTGATGGGCGGTTTCTCCCCCCACGCTCCGTTCTTCGCCGCGGCAGCCTTGAACGGCCTCAATTTCCTGACGGGCTGTTTCCTTTTGCCGGAGTCGCACAAAGGCGAACGCCGGCCGTTACGCCGGGAGGCTCTCAACCCGCTCGCTTCGTTCCGGTGGGCCCGGGGCATGACCGTCGTCGCCGCCCTGATGGCGGTCTTCTTCATCATGCAACTTGTCGGACAGGTGCCGGCCGCGCTTTGGGTCATTTTCGGCGAGGATCGCTTTCACTGGGACGCGACCACGATCGGCATTTCGCTTGCCGCATTTGGCATTCTGCATTCACTCGCCCAGGCAATGATCACCGGCCCTGTAGCCGCCCGGCTCGGCGAAAGGCGGGCACTCATGCTCGGAATGATTGCCGACGGCACAGGCTACATCCTGCTTGCCTTCGCGACACGGGGATGGATGGCGTTCCCGATCATGGTCCTGCTTGCTTCGGGTGGCATCGGAATGCCGGCGCTGCAGCATGTGTTT | [CP083586](https://www.ncbi.nlm.nih.gov/nuccore/CP083586) | 99.67 |
|  | NMJ7 | GTGGCAGTTCGCTCGGCTGATTATGCCGGTGCTGCCGGGCCTCCTGCGCGATCTGGTTCACTCGAACGACGTCACCGCCCACTATGGCATTCTGCTGGCGCTGTATGCGTTGATGCAATTTGCCTGCGCACCTGTGCTGGGCGCGCTGTCGGATCGTTTCGGGCGGCGGCCGGTCTTGCTCGTCTCGCTGGCCGGCGCTGCTGTCGACTACGCCATCATGGCGACGGCGCCTTTCCTTTGGGTTCTCTATATCGGGCGGATCGTGGCCGGCATCACCGGGGCGACTGGGGCGGTAGCCGGCGCTTATATTGCCGATATCACTGATGGCGATGAGCGCGCGCGGCACTTCGGCTTCATGAGCGCCTGTTTCGGGTTCGGGATGGTCGCGGGACCTGTGCTCGGTGGGCTGATGGGCGGTTTCTCCCCCCACGCTCCGTTCTTCGCCGCGGCAGCCTTGAACGGCCTCAATTTCCTGACGGGCTGTTTCCTTTTGCCGGAGTCGCACAAAGGCGAACGCCGGCCGTTACGCCGGGAGGCTCTCAACCCGCTCGCTTCGTTCCGGTGGGCCCGGGGCATGACCGTCGTCGCCGCCCTGATGGCGGTCTTCTTCATCATGCAACTTGTCGGACAGGTGCCGGCCGCGCTTTGGGTCATTTTCGGCGAGGATCGCTTTCACTGGGACGCGACCACGATCGGCATTTCGCTTGCCGCATTTGGCATTCTGCATTCACTCGCCCAGGCAATGATCACCGGCCCTGTAGCCGCCCGGCTCGGCGAAAGGCGGGCACTCATGCTCGGAATGATTGCCGACGGCACAGGCTACATCCTGCTTGCCTTCGCGACACGGGGATGGATGGCGTTCCCGATCATGGTCCTGCTTGCTTCGGGTGGCATCGGAATGCCGGCGCTGCAGCATG | [MZ475699](https://www.ncbi.nlm.nih.gov/nuccore/MZ475699) | 99.78 |
|  | NM15 | GTGCAGTTGCTCGGCTGATTATGCCGGTGCTGCCGGGCCTCCTGCGCGATCTGGTTCACTCGAACGACGTCACCGCCCACTATGGCATTCTGCTGGCGCTGTATGCGTTGATGCAATTTGCCTGCGCACCTGTGCTGGGCGCGCTGTCGGATCGTTTCGGGCGGCGGCCGGTCTTGCTCGTCTCGCTGGCCGGCGCTGCTGTCGACTACGCCATCATGGCGACGGCGCCTTTCCTTTGGGTTCTCTATATCGGGCGGATCGTGGCCGGCATCACCGGGGCGACTGGGGCGGTAGCCGGCGCTTATATTGCCGATATCACTGATGGCGATGAGCGCGCGCGGCACTTCGGCTTCATGAGCGCCTGTTTCGGGTTCGGGATGGTCGCGGGACCTGTGCTCGGTGGGCTGATGGGCGGTTTCTCCCCCCACGCTCCGTTCTTCGCCGCGGCAGCCTTGAACGGCCTCAATTTCCTGACGGGCTGTTTCCTTTTGCCGGAGTCGCACAAAGGCGAACGCCGGCCGTTACGCCGGGAGGCTCTCAACCCGCTCGCTTCGTTCCGGTGGGCCCGGGGCATGACCGTCGTCGCCGCCCTGATGGCGGTCTTCTTCATCATGCAACTTGTCGGACAGGTGCCGGCCGCGCTTTGGGTCATTTTCGGCGAGGATCGCTTTCACTGGGACGCGACCACGATCGGCATTTCGCTTGCCGCATTTGGCATTCTGCATTCACTCGCCCAGGCAATGATCACCGGCCCTGTAGCCGCCCGGCTCGGCGAAAGGCGGGCACTCATGCTCGGAATGATTGCCGACGGCACAGGCTACATCCTGCTTGCCTTCGCGACACGGGGATGGATGGCGTTCCCGATCATGGTCCTGCTTGCTTCGGGTGGCATCGGAATGCCGGCGCTGCAGCAGT | [MZ475699](https://www.ncbi.nlm.nih.gov/nuccore/MZ475699) | 99.78 |
|  | NM22 | GTGGCAGTTCGCTCGGCTGATTATGCCGGTGCTGCCGGGCCTCCTGCGCGATCTGGTTCACTCGAACGACGTCACCGCCCACTATGGCATTCTGCTGGCGCTGTATGCGTTGATGCAATTTGCCTGCGCACCTGTGCTGGGCGCGCTGTCGGATCGTTTCGGGCGGCGGCCGGTCTTGCTCGTCTCGCTGGCCGGCGCTGCTGTCGACTACGCCATCATGGCGACGACGCCTTTCCTTTGGGTTCTCTATATCGGGCGGATCGTGGCCGGCATCACCGGGGCGACTGGGGCGGTAGCCGGCGCTTATATTGCCGATATCACTGATGGCGATGAGCGCGCGCGGCACTTCGGCTTCATGAGCGCCTGTTTCGGGTTCGGGATGGTCGCGGGACCTGTGCTCGGTGGGCTGATGGGCGGTTTCTCCCCCCACGCTCCGTTCTTCGCCGCGGCAGCCTTGAACGGCCTCAATTTCCTGACGGGCTGTTTCCTTTTGCCGGAGTCGCACAAAGGCGAACGCCGGCCGTTACGCCGGGAGGCTCTCAACCCGCTCGCTTCGTTCCGGTGGGCCCGGGGCATGACCGTCGTCGCCGCCCTGATGGCGGTCTTCTTCATCATGCAACTTGTCGGACAGGTGCCGGCCGCGCTTTGGGTCATTTTCGGCGAGGATCGCTTTCACTGGGACGCGACCACGATCGGCATTTCGCTTGCCGCATTTGGCATTCTGCATTCACTCGCCCAGGCAATGATCACCGGCCCTGTAGCCGCCCGGCTCGGCGAAAGGCGGGCACTCATGCTCGGAATGATTGCCGACGGCACAGGCTACATCCTGCTTGCCTTCGCGACACGGGGATGGATGGCGTTCCCGATCATGGTCCTGCTTGCTTCGGGTGGCATCGGAATGCCGGCGCTGCAGATGTG | [CP083586](https://www.ncbi.nlm.nih.gov/nuccore/CP083586) | 99.89 |
|  | NM34 | GAGCCAGTTCGGATCGGCTGATTATGCCGGTGCTGCCGGGCCTCCTGCGCGATCTGGTTCACTCGAACGACGTCACCGCCCACTATGGCATTCTGCTGGCGCTGTATGCGTTGATGCAATTTGCCTGCGCACCTGTGCTGGGCGCGCTGTCGGATCGTTTCGGGCGGCGGCCGGTCTTGCTCGTCTCGCTGGCCGGCGCTGCTGTCGACTACGCCATCATGGCGACGGCGCCTTTCCTTTGGGTTCTCTATATCGGGCGGATCGTGGCCGGCATCACCGGGGCGACTGGGGCGGTAGCCGGCGCTTATATTGCCGATATCACTGATGGCGATGAGCGCGCGCGGCACTTCGGCTTCATGAGCGCCTGTTTCGGGTTCGGGATGGTCGCGGGACCTGTGCTCGGTGGGCTGATGGGCGGTTTCTCCCCCCACGCTCCGTTCTTCGCCGCGGCAGCCTTGAACGGCCTCAATTTCCTGACGGGCTGTTTCCTTTTGCCGGAGTCGCACAAAGGCGAACGCCGGCCGTTACGCCGGGAGGCTCTCAACCCGCTCGCTTCGTTCCGGTGGGCCCGGGGCATGACCGTCGTCGCCGCCCTGATGGCGGTCTTCTTCATCATGCAACTTGTCGGACAGGTGCCGGCCGCGCTTTGGGTCATTTTCGGCGAGGATCGCTTTCACTGGGACGCGACCACGATCGGCATTTCGCTTGCCGCATTTGGCATTCTGCATTCACTCGCCCAGGCAATGATCACCGGCCCTGTAGCCGCCCGGCTCGGCGAAAGGCGGGCACTCATGCTCGGAATGATTGCCGACGGCACAGGCTACATCCTGCTTGCCTTCGCGACACGGGGATGGATGGCGTTCCCGATCATGGTCCTGCTTGCTTCGGGTGGCATCGGAATGCCGGCGCTGCAAGCATTGTT | [MZ475699](https://www.ncbi.nlm.nih.gov/nuccore/MZ475699) | 99.67 |
|  | GN49 | ATGCAGTCGCTCGGCTGATTATGCCGGTGCTGCCGGGCCTCCTGCGCGATCTGGTTCACTCGAACGACGTCACCGCCCACTATGGCATTCTGCTGGCGCTGTATGCGTTGATGCAATTTGCCTGCGCACCTGTGCTGGGCGCGCTGTCGGATCGTTTCGGGCGGCGGCCGGTCTTGCTCGTCTCGCTGGCCGGCGCTGCTGTCGACTACGCCATCATGGCGACGGCGCCTTTCCTTTGGGTTCTCTATATCGGGCGGATCGTGGCCGGCATCACCGGGGCGACTGGGGCGGTAGCCGGCGCTTATATTGCCGATATCACTGATGGCGATGAGCGCGCGCGGCACTTCGGCTTCATGAGCGCCTGTTTCGGGTTCGGGATGGTCGCGGGACCTGTGCTCGGTGGGCTGATGGGCGGTTTCTCCCCCCACGCTCCGTTCTTCGCCGCGGCAGCCTTGAACGGCCTCAATTTCCTGACGGGCTGTTTCCTTTTGCCGGAGTCGCACAAAGGCGAACGCCGGCCGTTACGCCGGGAGGCTCTCAACCCGCTCGCTTCGTTCCGGTGGGCCCGGGGCATGACCGTCGTCGCCGCCCTGATGGCGGTCTTCTTCATCATGCAACTTGTCGGACAGGTGCCGGCCGCGCTTTGGGTCATTTTCGGCGAGGATCGCTTTCACTGGGACGCGACCACGATCGGCATTTCGCTTGCCGCATTTGGCATTCTGCATTCACTCGCCCAGGCAATGATCACCGGCCCTGTAGCCGCCCGGCTCGGCGAAAGGCGGGCACTCATGCTCGGAATGATTGCCGACGGCACAGGCTACATCCTGCTTGCCTTCGCGACACGGGGATGGATGGCGTTCCCGATCATGGTCCTGCTTGCTTCGGGTGGCATCGGAATGCCGGCGCTGCAGCAGT | [MZ475699](https://www.ncbi.nlm.nih.gov/nuccore/MZ475699) | 99.45 |
|  | GN57 | GTGGCATTGGCTCGGCTGATTATGCCGGTGCTGCCGGGCCTCCTGCGCGATCTGGTTCACTCGAACGACGTCACCGCCCACTATGGCATTCTGCTGGCGCTGTATGCGTTGATGCAATTTGCCTGCGCACCTGTGCTGGGCGCGCTGTCGGATCGTTTCGGGCGGCGGCCGGTCTTGCTCGTCTCGCTGGCCGGCGCTGCTGTCGACTACGCCATCATGGCGACGGCGCCTTTCCTTTGGGTTCTCTATATCGGGCGGATCGTGGCCGGCATCACCGGGGCGACTGGGGCGGTAGCCGGCGCTTATATTGCCGATATCACTGATGGCGATGAGCGCGCGCGGCACTTCGGCTTCATGAGCGCCTGTTTCGGGTTCGGGATGGTCGCGGGACCTGTGCTCGGTGGGCTGATGGGCGGTTTCTCCCCCCACGCTCCGTTCTTCGCCGCGGCAGCCTTGAACGGCCTCAATTTCCTGACGGGCTGTTTCCTTTTGCCGGAGTCGCACAAAGGCGAACGCCGGCCGTTACGCCGGGAGGCTCTCAACCCGCTCGCTTCGTTCCGGTGGGCCCGGGGCATGACCGTCGTCGCCGCCCTGATGGCGGTCTTCTTCATCATGCAACTTGTCGGACAGGTGCCGGCCGCGCTTTGGGTCATTTTCGGCGAGGATCGCTTTCACTGGGACGCGACCACGATCGGCATTTCGCTTGCCGCATTTGGCATTCTGCATTCACTCGCCCAGGCAATGATCACCGGCCCTGTAGCCGCCCGGCTCGGCGAAAGGCGGGCACTCATGCTCGGAATGATTGCCGACGGCACAGGCTACATCCTGCTTGCCTTCGCGACACGGGGATGGATGGCGTTCCCGATCATGGTCCTGCTTGCTTCGGGTGGCATCGGAATGCCGGCGCTGCAACATGTG | [CP086203](https://www.ncbi.nlm.nih.gov/nuccore/CP086203) | 99.56 |
|  | GN62 | GTGCAGTTCGCTCGGCTGATTATGCCGGTGCTGCCGGGCCTCCTGCGCGATCTGGTTCACTCGAACGACGTCACCGCCCACTATGGCATTCTGCTGGCGCTGTATGCGTTGATGCAATTTGCCTGCGCACCTGTGCTGGGCGCGCTGTCGGATCGTTTCGGGCGGCGGCCGGTCTTGCTCGTCTCGCTGGCCGGCGCTGCTGTCGACTACGCCATCATGGCGACGACGCCTTTCCTTTGGGTTCTCTATATCGGGCGGATCGTGGCCGGCATCACCGGGGCGACTGGGGCGGTAGCCGGCGCTTATATTGCCGATATCACTGATGGCGATGAGCGCGCGCGGCACTTCGGCTTCATGAGCGCCTGTTTCGGGTTCGGGATGGTCGCGGGACCTGTGCTCGGTGGGCTGATGGGCGGTTTCTCCCCCCACGCTCCGTTCTTCGCCGCGGCAGCCTTGAACGGCCTCAATTTCCTGACGGGCTGTTTCCTTTTGCCGGAGTCGCACAAAGGCGAACGCCGGCCGTTACGCCGGGAGGCTCTCAACCCGCTCGCTTCGTTCCGGTGGGCCCGGGGCATGACCGTCGTCGCCGCCCTGATGGCGGTCTTCTTCATCATGCAACTTGTCGGACAGGTGCCGGCCGCGCTTTGGGTCATTTTCGGCGAGGATCGCTTTCACTGGGACGCGACCACGATCGGCATTTCGCTTGCCGCATTTGGCATTCTGCATTCACTCGCCCAGGCAATGATCACCGGCCCTGTAGCCGCCCGGCTCGGCGAAAGGCGGGCACTCATGCTCGGAATGATTGCCGACGGCACAGGCTACATCCTGCTTGCCTTCGCGACACGGGGATGGATGGCGTTCCCGATCATGGTCCTGCTTGCTTCGGGTGGCATCGGAATGCCGGCGCTGCAGATGTG | [CP083586](https://www.ncbi.nlm.nih.gov/nuccore/CP083586) | 99.89 |
|  | GN64 | CGGCAGTTCGCTCGGCTGATTATGCCGGTGCTGCCGGGCCTCCTGCGCGATCTGGTTCACTCGAACGACGTCACCGCCCACTATGGCATTCTGCTGGCGCTGTATGCGTTGATGCAATTTGCCTGCGCACCTGTGCTGGGCGCGCTGTCGGATCGTTTCGGGCGGCGGCCGGTCTTGCTCGTCTCGCTGGCCGGCGCTGCTGTCGACTACGCCATCATGGCGACGGCGCCTTTCCTTTGGGTTCTCTATATCGGGCGGATCGTGGCCGGCATCACCGGGGCGACTGGGGCGGTAGCCGGCGCTTATATTGCCGATATCACTGATGGCGATGAGCGCGCGCGGCACTTCGGCTTCATGAGCGCCTGTTTCGGGTTCGGGATGGTCGCGGGACCTGTGCTCGGTGGGCTGATGGGCGGTTTCTCCCCCCACGCTCCGTTCTTCGCCGCGGCAGCCTTGAACGGCCTCAATTTCCTGACGGGCTGTTTCCTTTTGCCGGAGTCGCACAAAGGCGAACGCCGGCCGTTACGCCGGGAGGCTCTCAACCCGCTCGCTTCGTTCCGGTGGGCCCGGGGCATGACCGTCGTCGCCGCCCTGATGGCGGTCTTCTTCATCATGCAACTTGTCGGACAGGTGCCGGCCGCGCTTTGGGTCATTTTCGGCGAGGATCGCTTTCACTGGGACGCGACCACGATCGGCATTTCGCTTGCCGCATTTGGCATTCTGCATTCACTCGCCCAGGCAATGATCACCGGCCCTGTAGCCGCCCGGCTCGGCGAAAGGCGGGCACTCATGCTCGGAATGATTGCCGACGGCACAGGCTACATCCTGCTTGCCTTCGCGACACGGGGATGGATGGCGTTCCCGATCATGGTCCTGCTTGCTTCGGGTGGCATCGGAATGCCGGCGCTGCAGAGTG | [MZ475699](https://www.ncbi.nlm.nih.gov/nuccore/MZ475699) | 99.89 |
|  | GN65 | CGGCGGTTCGGCTCGGCTGATTATGCCGGTGCTGCCGGGCCTCCTGCGCGATCTGGTTCACTCGAACGACGTCACCGCCCACTATGGCATTCTGCTGGCGCTGTATGCGTTGATGCAATTTGCCTGCGCACCTGTGCTGGGCGCGCTGTCGGATCGTTTCGGGCGGCGGCCGGTCTTGCTCGTCTCGCTGGCCGGCGCTGCTGTCGACTACGCCATCATGGCGACGACGCCTTTCCTTTGGGTTCTCTATATCGGGCGGATCGTGGCCGGCATCACCGGGGCGACTGGGGCGGTAGCCGGCGCTTATATTGCCGATATCACTGATGGCGATGAGCGCGCGCGGCACTTCGGCTTCATGAGCGCCTGTTTCGGGTTCGGGATGGTCGCGGGACCTGTGCTCGGTGGGCTGATGGGCGGTTTCTCCCCCCACGCTCCGTTCTTCGCCGCGGCAGCCTTGAACGGCCTCAATTTCCTGACGGGCTGTTTCCTTTTGCCGGAGTCGCACAAAGGCGAACGCCGGCCGTTACGCCGGGAGGCTCTCAACCCGCTCGCTTCGTTCCGGTGGGCCCGGGGCATGACCGTCGTCGCCGCCCTGATGGCGGTCTTCTTCATCATGCAACTTGTCGGACAGGTGCCGGCCGCGCTTTGGGTCATTTTCGGCGAGGATCGCTTTCACTGGGACGCGACCACGATCGGCATTTCGCTTGCCGCATTTGGCATTCTGCATTCACTCGCCCAGGCAATGATCACCGGCCCTGTAGCCGCCCGGCTCGGCGAAAGGCGGGCACTCATGCTCGGAATGATTGCCGACGGCACAGGCTACATCCTGCTTGCCTTCGCGACACGGGGATGGATGGCGTTCCCGATCATGGTCCTGCTTGCTTCGGGTGGCATCGGAATGCCGGCGCTGCAAGCATGT | [CP083586](https://www.ncbi.nlm.nih.gov/nuccore/CP083586) | 99.67 |
|  | GN67 | GTGCAGTTCGCTCGGCTGATTATGCCGGTGCTGCCGGGCCTCCTGCGCGATCTGGTTCACTCGAACGACGTCACCGCCCACTATGGCATTCTGCTGGCGCTGTATGCGTTGATGCAATTTGCCTGCGCACCTGTGCTGGGCGCGCTGTCGGATCGTTTCGGGCGGCGGCCGGTCTTGCTCGTCTCGCTGGCCGGCGCTGCTGTCGACTACGCCATCATGGCGACGGCGCCTTTCCTTTGGGTTCTCTATATCGGGCGGATCGTGGCCGGCATCACCGGGGCGACTGGGGCGGTAGCCGGCGCTTATATTGCCGATATCACTGATGGCGATGAGCGCGCGCGGCACTTCGGCTTCATGAGCGCCTGTTTCGGGTTCGGGATGGTCGCGGGACCTGTGCTCGGTGGGCTGATGGGCGGTTTCTCCCCCCACGCTCCGTTCTTCGCCGCGGCAGCCTTGAACGGCCTCAATTTCCTGACGGGCTGTTTCCTTTTGCCGGAGTCGCACAAAGGCGAACGCCGGCCGTTACGCCGGGAGGCTCTCAACCCGCTCGCTTCGTTCCGGTGGGCCCGGGGCATGACCGTCGTCGCCGCCCTGATGGCGGTCTTCTTCATCATGCAACTTGTCGGACAGGTGCCGGCCGCGCTTTGGGTCATTTTCGGCGAGGATCGCTTTCACTGGGACGCGACCACGATCGGCATTTCGCTTGCCGCATTTGGCATTCTGCATTCACTCGCCCAGGCAATGATCACCGGCCCTGTAGCCGCCCGGCTCGGCGAAAGGCGGGCACTCATGCTCGGAATGATTGCCGACGGCACAGGCTACATCCTGCTTGCCTTCGCGACACGGGGATGGATGGCGTTCCCGATCATGGTCCTGCTTGCTTCGGGTGGCATCGGAATGCCGGCGCTGCAGATGT | [AP025221](https://www.ncbi.nlm.nih.gov/nuccore/AP025221) | 99.89 |
|  | GN68 | CGCAAGTTCGGCTCGGCTGATTATGCCGGTGCTGCCGGGCCTCCTGCGCGATCTGGTTCACTCGAACGACGTCACCGCCCACTATGGCATTCTGCTGGCGCTGTATGCGTTGATGCAATTTGCCTGCGCACCTGTGCTGGGCGCGCTGTCGGATCGTTTCGGGCGGCGGCCGGTCTTGCTCGTCTCGCTGGCCGGCGCTGCTGTCGACTACGCCATCATGGCGACGACGCCTTTCCTTTGGGTTCTCTATATCGGGCGGATCGTGGCCGGCATCACCGGGGCGACTGGGGCGGTAGCCGGCGCTTATATTGCCGATATCACTGATGGCGATGAGCGCGCGCGGCACTTCGGCTTCATGAGCGCCTGTTTCGGGTTCGGGATGGTCGCGGGACCTGTGCTCGGTGGGCTGATGGGCGGTTTCTCCCCCCACGCTCCGTTCTTCGCCGCGGCAGCCTTGAACGGCCTCAATTTCCTGACGGGCTGTTTCCTTTTGCCGGAGTCGCACAAAGGCGAACGCCGGCCGTTACGCCGGGAGGCTCTCAACCCGCTCGCTTCGTTCCGGTGGGCCCGGGGCATGACCGTCGTCGCCGCCCTGATGGCGGTCTTCTTCATCATGCAACTTGTCGGACAGGTGCCGGCCGCGCTTTGGGTCATTTTCGGCGAGGATCGCTTTCACTGGGACGCGACCACGATCGGCATTTCGCTTGCCGCATTTGGCATTCTGCATTCACTCGCCCAGGCAATGATCACCGGCCCTGTAGCCGCCCGGCTCGGCGAAAGGCGGGCACTCATGCTCGGAATGATTGCCGACGGCACAGGCTACATCCTGCTTGCCTTCGCGACACGGGGATGGATGGCGTTCCCGATCATGGTCCTGCTTGCTTCGGGTGGCATCGGAATGCCGGCGCTGCAGATGTG | [CP083586](https://www.ncbi.nlm.nih.gov/nuccore/CP083586) | 100.00 |
|  | dc60 | GGGCATTTGGATCGGCTGATTATGCCGGTGCTGCCGGGCCTCCTGCGCGATCTGGTTCACTCGAACGACGTCGCCGCCCACTATGGCATTCTGCTGGCGCTGTATGCGTTGATGCAATTTGCCTGCGCACCTGTGCTGGGCGCGCTGTCGGATCGTTTCGGGCGGCGGCCGGTCTTGCTCGTCTCGCTGGCCGGCGCTGCTGTCGACTACGCCATCATGGCGACGGCGCCTTTCCTTTGGGTTCTCTATATCGGGCGGATCGTGGCCGGCATCACCGGGGCGACTGGGGCGGTAGCCGGCGCTTATATTGCCGATATCACTGATGGCGATGAGCGCGCGCGGCACTTCGGCTTCATGAGCGCCTGTTTCGGGTTCGGGATGGTCGCGGGACCTGTGCTCGGTGGGCTGATGGGCGGTTTCTCCCCCCACGCTCCGTTCTTCGCCGCGGCAGCCTTGAACGGCCTCAATTTCCTGACGGGCTGTTTCCTTTTGCCGGAGTCGCACAAAGGCGAACGCCGGCCGTTACGCCGGGAGGCTCTCAACCCGCTCGCTTCGTTCCGGTGGGCCCGGGGCATGACCGTCGTCGCCGCCCTGATGGCGGTCTTCTTCATCATGCAACTTGTCGGACAGGTGCCGGCCGCGCTTTGGGTCATTTTCGGCGAGGATCGCTTTCACTGGGACGCGACCACGATCGGCATTTCGCTTGCCGCATTTGGCATTCTGCATTCACTCGCCCAGGCAATGATCACCGGCCCTGTAGCCGCCCGGCTCGGCGAAAGGCGGGCACTCATGCTCGGAATGATTGCCGACGGCACAGGCTACATCCTGCTTGCCTTCGCGACACGGGGATGGATGGCGTTCCCGATCATGGTCCTGCTTGCTTCGGGTGGCATCGGAATGCCGGCGCTGCAGATGTG | [CP055262](https://www.ncbi.nlm.nih.gov/nuccore/CP055262) | 99.89 |
|  | dc63 | GAGGAAATTGGCTCGGCTGATTATGCCGGTGCTGCCGGGCCTCCTGCGCGATCTGGTTCACTCGAACGACGTCACCGCCCACTATGGCATTCTGCTGGCGCTGTATGCGTTGATGCAATTTGCCTGCGCACCTGTGCTGGGCGCGCTGTCGGATCGTTTCGGGCGGCGGCCGGTCTTGCTCGTCTCGCTGGCCGGCGCTGCTGTCGACTACGCCATCATGGCGACGGCGCCTTTCCTTTGGGTTCTCTATATCGGGCGGATCGTGGCCGGCATCACCGGGGCGACTGGGGCGGTAGCCGGCGCTTATATTGCCGATATCACTGATGGCGATGAGCGCGCGCGGCACTTCGGCTTCATGAGCGCCTGTTTCGGGTTCGGGATGGTCGCGGGACCTGTGCTCGGTGGGCTGATGGGCGGTTTCTCCCCCCACGCTCCGTTCTTCGCCGCGGCAGCCTTGAACGGCCTCAATTTCCTGACGGGCTGTTTCCTTTTGCCGGAGTCGCACAAAGGCGAACGCCGGCCGTTACGCCGGGAGGCTCTCAACCCGCTCGCTTCGTTCCGGTGGGCCCGGGGCATGACCGTCGTCGCCGCCCTGATGGCGGTCTTCTTCATCATGCAACTTGTCGGACAGGTGCCGGCCGCGCTTTGGGTCATTTTCGGCGAGGATCGCTTTCACTGGGACGCGACCACGATCGGCATTTCGCTTGCCGCATTTGGCATTCTGCATTCACTCGCCCAGGCAATGATCACCGGCCCTGTAGCCGCCCGGCTCGGCGAAAGGCGGGCACTCATGCTCGGAATGATTGCCGACGGCACAGGCTACATCCTGCTTGCCTTCGCGACACGGGGATGGATGGCGTTCCCGATCATGGTCCTGCTTGCTTCGGGTGGCATCGGAATGCCGGCGCTGCAGATGTG | [MZ475699](https://www.ncbi.nlm.nih.gov/nuccore/MZ475699) | 99.78 |
|  | dc227 | GGCGTTCGCTCGGCTGATTATGCCGGTGCTGCCGGGCCTCCTGCGCGATCTGGTTCACTCGAACGACGTCACCGCCCACTATGGCATTCTGCTGGCGCTGTATGCGTTGATGCAATTTGCCTGCGCACCTGTGCTGGGCGCGCTGTCGGATCGTTTCGGGCGGCGGCCGGTCTTGCTCGTCTCGCTGGCCGGCGCTGCTGTCGACTACGCCATCATGGCGACGGCGCCTTTCCTTTGGGTTCTCTATATCGGGCGGATCGTGGCCGGCATCACCGGGGCGACTGGGGCGGTAGCCGGCGCTTATATTGCCGATATCACTGATGGCGATGAGCGCGCGCGGCACTTCGGCTTCATGAGCGCCTGTTTCGGGTTCGGGATGGTCGCGGGACCTGTGCTCGGTGGGCTGATGGGCGGTTTCTCCCCCCACGCTCCGTTCTTCGCCGCGGCAGCCTTGAACGGCCTCAATTTCCTGACGGGCTGTTTCCTTTTGCCGGAGTCGCACAAAGGCGAACGCCGGCCGTTACGCCGGGAGGCTCTCAACCCGCTCGCTTCGTTCCGGTGGGCCCGGGGCATGACCGTCGTCGCCGCCCTGATGGCGGTCTTCTTCATCATGCAACTTGTCGGACAGGTGCCGGCCGCGCTTTGGGTCATTTTCGGCGAGGATCGCTTTCACTGGGACGCGACCACGATCGGCATTTCGCTTGCCGCATTTGGCATTCTGCATTCACTCGCCCAGGCAATGATCACCGGCCCTGTAGCCGCCCGGCTCGGCGAAAGGCGGGCACTCATGCTCGGAATGATTGCCGACGGCACAGGCTACATCCTGCTTGCCTTCGCGACACGGGGATGGATGGCGTTCCCGATCATGGTCCTGCTTGCTTCGGGTGGCATCGGAATGCCGGCGCTGCAGAGTG | [MZ475699](https://www.ncbi.nlm.nih.gov/nuccore/MZ475699) | 99.89 |
|  | dc275 | CGGCCAGTTCGCTCGGCTGATTATGCCGGTGCTGCCGGGCCTCCTGCGCGATCTGGTTCACTCGAACGACGTCACCGCCCACTATGGCATTCTGCTGGCGCTGTATGCGTTGATGCAATTTGCCTGCGCACCTGTGCTGGGCGCGCTGTCGGATCGTTTCGGGCGGCGGCCGGTCTTGCTCGTCTCGCTGGCCGGCGCTGCTGTCGACTACGCCATCATGGCGACGACGCCTTTCCTTTGGGTTCTCTATATCGGGCGGATCGTGGCCGGCATCACCGGGGCGACTGGGGCGGTAGCCGGCGCTTATATTGCCGATATCACTGATGGCGATGAGCGCGCGCGGCACTTCGGCTTCATGAGCGCCTGTTTCGGGTTCGGGATGGTCGCGGGACCTGTGCTCGGTGGGCTGATGGGCGGTTTCTCCCCCCACGCTCCGTTCTTCGCCGCGGCAGCCTTGAACGGCCTCAATTTCCTGACGGGCTGTTTCCTTTTGCCGGAGTCGCACAAAGGCGAACGCCGGCCGTTACGCCGGGAGGCTCTCAACCCGCTCGCTTCGTTCCGGTGGGCCCGGGGCATGACCGTCGTCGCCGCCCTGATGGCGGTCTTCTTCATCATGCAACTTGTCGGACAGGTGCCGGCCGCGCTTTGGGTCATTTTCGGCGAGGATCGCTTTCACTGGGACGCGACCACGATCGGCATTTCGCTTGCCGCATTTGGCATTCTGCATTCACTCGCCCAGGCAATGATCACCGGCCCTGTAGCCGCCCGGCTCGGCGAAAGGCGGGCACTCATGCTCGGAATGATTGCCGACGGCACAGGCTACATCCTGCTTGCCTTCGCGACACGGGGATGGATGGCGTTCCCGATCATGGTCCTGCTTGCTTCGGGTGGCATCGGAATGCCGGCGCTGCAAGCATTGT | [CP083586](https://www.ncbi.nlm.nih.gov/nuccore/CP083586) | 99.78 |
|  | S10 | GTGCAGTTCGCTCGGCTGATTATGCCGGTGCTGCCGGGCCTCCTGCGCGATCTGGTTCACTCGAACGACGTCACCGCCCACTATGGCATTCTGCTGGCGCTGTATGCGTTGATGCAATTTGCCTGCGCACCTGTGCTGGGCGCGCTGTCGGATCGTTTCGGGCGGCGGCCGGTCTTGCTCGTCTCGCTGGCCGGCGCTGCTGTCGACTACGCCATCATGGCGACGGCGCCTTTCCTTTGGGTTCTCTATATCGGGCGGATCGTGGCCGGCATCACCGGGGCGACTGGGGCGGTAGCCGGCGCTTATATTGCCGATATCACTGATGGCGATGAGCGCGCGCGGCACTTCGGCTTCATGAGCGCCTGTTTCGGGTTCGGGATGGTCGCGGGACCTGTGCTCGGTGGGCTGATGGGCGGTTTCTCCCCCCACGCTCCGTTCTTCGCCGCGGCAGCCTTGAACGGCCTCAATTTCCTGACGGGCTGTTTCCTTTTGCCGGAGTCGCACAAAGGCGAACGCCGGCCGTTACGCCGGGAGGCTCTCAACCCGCTCGCTTCGTTCCGGTGGGCCCGGGGCATGACCGTCGTCGCCGCCCTGATGGCGGTCTTCTTCATCATGCAACTTGTCGGACAGGTGCCGGCCGCGCTTTGGGTCATTTTCGGCGAGGATCGCTTTCACTGGGACGCGACCACGATCGGCATTTCGCTTGCCGCATTTGGCATTCTGCATTCACTCGCCCAGGCAATGATCACCGGCCCTGTAGCCGCCCGGCTCGGCGAAAGGCGGGCACTCATGCTCGGAATGATTGCCGACGGCACAGGCTACATCCTGCTTGCCTTCGCGACACGGGGATGGATGGCGTTCCCGATCATGGTCCTGCTTGCTTCGGGTGGCATCGGAATGCCGGCGCTGCAGAGTGT | [MZ475699](https://www.ncbi.nlm.nih.gov/nuccore/MZ475699) | 99.89 |
|  | Z25 | GGGGCGTTCGCATCGGCTGATTATGCCGGTGCTGCCGGGCCTCCTGCGCGATCTGGTTCACTCGAACGACGTCACCGCCCACTATGGCATTCTGCTGGCGCTGTATGCGTTGATGCAATTTGCCTGCGCACCTGTGCTGGGCGCGCTGTCGGATCGTTTCGGGCGGCGGCCGGTCTTGCTCGTCTCGCTGGCCGGCGCTGCTGTCGACTACGCCATCATGGCGACGACGCCTTTCCTTTGGGTTCTCTATATCGGGCGGATCGTGGCCGGCATCACCGGGGCGACTGGGGCGGTAGCCGGCGCTTATATTGCCGATATCACTGATGGCGATGAGCGCGCGCGGCACTTCGGCTTCATGAGCGCCTGTTTCGGGTTCGGGATGGTCGCGGGACCTGTGCTCGGTGGGCTGATGGGCGGTTTCTCCCCCCACGCTCCGTTCTTCTCCGCGGCAGCCTTGAACGGCCTCAATTTCCTGACGGGCTGTTTCCTTTTGCCGGAGTCGCACAAAGGCGAACGCCGGCCGTTACGCCGGGAGGCTCTCAACCCGCTCGCTTCGTTCCGGTGGGCCCGGGGCATGACCGTCGTCGCCGCCCTGATGGCGGTCTTCTTCATCATGCAACTTGTCGGACAGGTGCCGGCCGCGCTTTGGGTCATTTTCGGCGAGGATCGCTTTCACTGGGACGCGACCACGATCGGCATTTCGCTTGCCGCATTTGGCATTCTGCATTCACTCGCCCAGGCAATGATCACCGGCCCTGTAGCCGCCCGGCTCGGCGAAAGGCGGGCACTCATGCTCGGAATGATTGCCGACGGCACAGGCTACATCCTGCTTGCCTTCGCGACACGGGGATGGATGGCGTTCCCGATCATGGTCCTGCTTGCTTCGGGTGGCATCGGAATGCCGGCGCTGCAGCATGTG | [CP083586](https://www.ncbi.nlm.nih.gov/nuccore/CP083586) | 99.56 |
|  | YO-5 | GGGCAGTTCGGCACGGCTGATTATGCCGGTGCTGCCGGGCCTCCTGCGCGATCTGGTTCACTCGAACGACGTCACCGCCCACTATGGCATTCTGCTGGCGCTGTATGCGTTGATGCAATTTGCCTGCGCACCTGTGCTGGGCGCGCTGTCGGATCGTTTCGGGCGGCGGCCGGTCTTGCTCGTCTCGCTGGCCGGCGCTGCTGTCGACTACGCCATCATGGCGACGACGCCTTTCCTTTGGGTTCTCTATATCGGGCGGATCGTGGCCGGCATCACCGGGGCGACTGGGGCGGTAGCCGGCGCTTATATTGCCGATATCACTGATGGCGATGAGCGCGCGCGGCACTTCGGCTTCATGAGCGCCTGTTTCGGGTTCGGGATGGTCGCGGGACCTGTGCTCGGTGGGCTGATGGGCGGTTTCTCCCCCCACGCTCCGTTCTTCGCCGCGGCAGCCTTGAACGGCCTCAATTTCCTGACGGGCTGTTTCCTTTTGCCGGAGTCGCACAAAGGCGAACGCCGGCCGTTACGCCGGGAGGCTCTCAACCCGCTCGCTTCGTTCCGGTGGGCCCGGGGCATGACCGTCGTCGCCGCCCTGATGGCGGTCTTCTTCATCATGCAACTTGTCGGACAGGTGCCGGCCGCGCTTTGGGTCATTTTCGGCGAGGATCGCTTTCACTGGGACGCGACCACGATCGGCATTTCGCTTGCCGCATTTGGCATTCTGCATTCACTCGCCCAGGCAATGATCACCGGCCCTGTAGCCGCCCGGCTCGGCGAAAGGCGGGCACTCATGCTCGGAATGATTGCCGACGGCACAGGCTACATCCTGCTTGCCTTCGCGACACGGGGATGGATGGCGTTCCCGATCATGGTCCTGCTTGCTTCGGGTGGCATCGGAATGCCGGCGCTGCAGCATGTG | [CP083586](https://www.ncbi.nlm.nih.gov/nuccore/CP083586) | 99.56 |
|  | YO-7 | GTGGCAGTTCGCTCGGCTGATTATGCCGGTGCTGCCGGGCCTCCTGCGCGATCTGGTTCACTCGAACGACGTCACCGCCCACTATGGCATTCTGCTGGCGCTGTATGCGTTGATGCAATTTGCCTGCGCACCTGTGCTGGGCGCGCTGTCGGATCGTTTCGGGCGGCGGCCGGTCTTGCTCGTCTCGCTGGCCGGCGCTGCTGTCGACTACGCCATCATGGCGACGACGCCTTTCCTTTGGGTTCTCTATATCGGGCGGATCGTGGCCGGCATCACCGGGGCGACTGGGGCGGTAGCCGGCGCTTATATTGCCGATATCACTGATGGCGATGAGCGCGCGCGGCACTTCGGCTTCATGAGCGCCTGTTTCGGGTTCGGGATGGTCGCGGGACCTGTGCTCGGTGGGCTGATGGGCGGTTTCTCCCCCCACGCTCCGTTCTTCGCCGCGGCAGCCTTGAACGGCCTCAATTTCCTGACGGGCTGTTTCCTTTTGCCGGAGTCGCACAAAGGCGAACGCCGGCCGTTACGCCGGGAGGCTCTCAACCCGCTCGCTTCGTTCCGGTGGGCCCGGGGCATGACCGTCGTCGCCGCCCTGATGGCGGTCTTCTTCATCATGCAACTTGTCGGACAGGTGCCGGCCGCGCTTTGGGTCATTTTCGGCGAGGATCGCTTTCACTGGGACGCGACCACGATCGGCATTTCGCTTGCCGCATTTGGCATTCTGCATTCACTCGCCCAGGCAATGATCACCGGCCCTGTAGCCGCCCGGCTCGGCGAAAGGCGGGCACTCATGCTCGGAATGATTGCCGACGGCACAGGCTACATCCTGCTTGCCTTCGCGACACGGGGATGGATGGCGTTCCCGATCATGGTCCTGCTTGCTTCGGGTGGCATCGGAATGCCGGCGCTGCAGAGTG | [CP083586](https://www.ncbi.nlm.nih.gov/nuccore/CP083586) | 99.56 |
|  | YO-9 | CAGCCGGTTCGGATCGGCTGATTATGCCGGTGCTGCCGGGCCTCCTGCGCGATCTGGTTCACTCGAACGACGTCACCGCCCACTATGGCATTCTGCTGGCGCTGTATGCGTTGATGCAATTTGCCTGCGCACCTGTGCTGGGCGCGCTGTCGGATCGTTTCGGGCGGCGGCCGGTCTTGCTCGTCTCGCTGGCCGGCGCTGCTGTCGACTACGCCATCATGGCGACGACGCCTTTCCTTTGGGTTCTCTATATCGGGCGGATCGTGGCCGGCATCACCGGGGCGACTGGGGCGGTAGCCGGCGCTTATATTGCCGATATCACTGATGGCGATGAGCGCGCGCGGCACTTCGGCTTCATGAGCGCCTGTTTCGGGTTCGGGATGGTCGCGGGACCTGTGCTCGGTGGGCTGATGGGCGGTTTCTCCCCCCACGCTCCGTTCTTCGCCGCGGCAGCCTTGAACGGCCTCAATTTCCTGACGGGCTGTTTCCTTTTGCCGGAGTCGCACAAAGGCGAACGCCGGCCGTTACGCCGGGAGGCTCTCAACCCGCTCGCTTCGTTCCGGTGGGCCCGGGGCATGACCGTCGTCGCCGCCCTGATGGCGGTCTTCTTCATCATGCAACTTGTCGGACAGGTGCCGGCCGCGCTTTGGGTCATTTTCGGCGAGGATCGCTTTCACTGGGACGCGACCACGATCGGCATTTCGCTTGCCGCATTTGGCATTCTGCATTCACTCGCCCAGGCAATGATCACCGGCCCTGTAGCCGCCCGGCTCGGCGAAAGGCGGGCACTCATGCTCGGAATGATTGCCGACGGCACAGGCTACATCCTGCTTGCCTTCGCGACACGGGGATGGATGGCGTTCCCGATCATGGTCCTGCTTGCTTCGGGTGGCATCGGAATGCCGGCGCTGCAGCATGT | [CP083586](https://www.ncbi.nlm.nih.gov/nuccore/CP083586) | 99.56 |
|  | YO-11 | CTGCAAGTTCGCTCGGCTGATTATGCCGGTGCTGCCGGGCCTCCTGCGCGATCTGGTTCACTCGAACGACGTCACCGCCCACTATGGCATTCTGCTGGCGCTGTATGCGTTGATGCAATTTGCCTGCGCACCTGTGCTGGGCGCGCTGTCGGATCGTTTCGGGCGGCGGCCGGTCTTGCTCGTCTCGCTGGCCGGCGCTGCTGTCGACTACGCCATCATGGCGACGACGCCTTTCCTTTGGGTTCTCTATATCGGGCGGATCGTGGCCGGCATCACCGGGGCGACTGGGGCGGTAGCCGGCGCTTATATTGCCGATATCACTGATGGCGATGAGCGCGCGCGGCACTTCGGCTTCATGAGCGCCTGTTTCGGGTTCGGGATGGTCGCGGGACCTGTGCTCGGTGGGCTGATGGGCGGTTTCTCCCCCCACGCTCCGTTCTTCGCCGCGGCAGCCTTGAACGGCCTCAATTTCCTGACGGGCTGTTTCCTTTTGCCGGAGTCGCACAAAGGCGAACGCCGGCCGTTACGCCGGGAGGCTCTCAACCCGCTCGCTTCGTTCCGGTGGGCCCGGGGCATGACCGTCGTCGCCGCCCTGATGGCGGTCTTCTTCATCATGCAACTTGTCGGACAGGTGCCGGCCGCGCTTTGGGTCATTTTCGGCGAGGATCGCTTTCACTGGGACGCGACCACGATCGGCATTTCGCTTGCCGCATTTGGCATTCTGCATTCACTCGCCCAGGCAATGATCACCGGCCCTGTAGCCGCCCGGCTCGGCGAAAGGCGGGCACTCATGCTCGGAATGATTGCCGACGGCACAGGCTACATCCTGCTTGCCTTCGCGACACGGGGATGGATGGCGTTCCCGATCATGGTCCTGCTTGCTTCGGGTGGCATCGGAATGCCGGCGCTGCAAGCATGGTT | [CP084534](https://www.ncbi.nlm.nih.gov/nuccore/CP084534) | 99.89 |
|  | YO-55 | ATGCAGTTCGCATCGGCTGATTATGCCGGTGCTGCCGGGCCTCCTGCGCGATCTGGTTCACTCGAACGACGTCACCGCCCACTATGGCATTCTGCTGGCGCTGTATGCGTTGATGCAATTTGCCTGCGCACCTGTGCTGGGCGCGCTGTCGGATCGTTTCGGGCGGCGGCCGGTCTTGCTCGTCTCGCTGGCCGGCGCTGCTGTCGACTACGCCATCATGGCGACGACGCCTTTCCTTTGGGTTCTCTATATCGGGCGGATCGTGGCCGGCATCACCGGGGCGACTGGGGCGGTAGCCGGCGCTTATATTGCCGATATCACTGATGGCGATGAGCGCGCGCGGCACTTCGGCTTCATGAGCGCCTGTTTCGGGTTCGGGATGGTCGCGGGACCTGTGCTCGGTGGGCTGATGGGCGGTTTCTCCCCCCACGCTCCGTTCTTCGCCGCGGCAGCCTTGAACGGCCTCAATTTCCTGACGGGCTGTTTCCTTTTGCCGGAGTCGCACAAAGGCGAACGCCGGCCGTTACGCCGGGAGGCTCTCAACCCGCTCGCTTCGTTCCGGTGGGCCCGGGGCATGACCGTCGTCGCCGCCCTGATGGCGGTCTTCTTCATCATGCAACTTGTCGGACAGGTGCCGGCCGCGCTTTGGGTCATTTTCGGCGAGGATCGCTTTCACTGGGACGCGACCACGATCGGCATTTCGCTTGCCGCATTTGGCATTCTGCATTCACTCGCCCAGGCAATGATCACCGGCCCTGTAGCCGCCCGGCTCGGCGAAAGGCGGGCACTCATGCTCGGAATGATTGCCGACGGCACAGGCTACATCCTGCTTGCCTTCGCGACACGGGGATGGATGGCGTTCCCGATCATGGTCCTGCTTGCTTCGGGTGGCATCGGAATGCCGGCGCTGCAGATGTG | [CP084534](https://www.ncbi.nlm.nih.gov/nuccore/CP084534) | 99.89 |
| *cmlA* | NMJ1 | GAAGAGCGCCATTGGGCGACACAGATACCGCTGTGGCGACACCAATACCCACTAGCCACATTGGAGCAATAAAGCCTAACACGGACTGCGAAGCCCATATTTCGGTGATGGCAAGCAATACTGCTCCAGCTATCAGGCATCCCATTCCCATTCGCAAGACACTTGGGCTGCCCCACTTGGGTATCACACGCCCCATAAAACGAGCCGTAAACACCATGGCAATTGCCACTGTGGCGAACAGCAGGCTGAAGCCAAGCTGAGACACACCTTGCCTGCCCATCATTAGTCCGGGCGCAATGGAGAAAAAGACGAAGAAGCTACCCATTCCAGCGGCGTAACACAACGTGTACAACCAGAAGTTCAGGCACTTAACGGGGAGTAGCAGCTGCGACCATTGCAAGCCCGCAACTCGTTGCACCCGGGTTTCAGGCCAGAATCGCCACGCTGCTGCAGATGCAGCGATCATGCCCAAACCTAGAAACGCAAAGATAGCCCGCCACCCAAGCCACATGTCGACGAGCGCTCCGAGCAATGGGCCTACCGCCGGGACCATGGCCAGCATGGATCCGAGTATGCCGTAAATGACATTACTTTCCTCGCGACCTGCGTAAATGTCACGTACTGTTGCAAATGTGGAAACAAGGCACGCCGAGGCACCACAAGCCTGAAGAATCCGAAGCCCCAGAAAGACTTCAGCCGATGACGTAAGAGCGAGGCCCATTGACGCCACAACGTAGGCGAGGCCACCTCCCAGTAGAACGGGGCGGCGCCCCAGTCGGTCCGATAGCGGTCCAAACAAGAGCTGACCGGCACCAATCATGACCAAGTACGTTGTCAGCGTAAGCTGAATTGTGCTCGCTGTCGTACCAAGCGCGTTTGGCATAAACGGCACTGCTGGCA | [AP022218](https://www.ncbi.nlm.nih.gov/nuccore/AP022218) | 100.00 |
|  | ZQ13 | TGCCAGCAGTGCCGTTTATGCCAAACGCGCTTGGTACGACAGCGAGCACAATTCAGCTTACGCTGACAACGTACTTGGTCATGATTGGTGCCGGTCAGCTCTTGTTTGGACCGCTATCGGACCGACTGGGGCGCCGCCCCGTTCTACTGGGAGGTGGCCTCGCCTACGTTGTGGCGTCAATGGGCCTCGCTCTTACGTCATCGGCTGAAGTCTTTCTGGGGCTTCGGATTCTTCAGGCTTGTGGTGCCTCGGCGTGCCTTGTTTCCACATTTGCAACAGTACGTGACATTTACGCAGGTCGCGAGGAAAGTAATGTCATTTACGGCATACTCGGATCCATGCTGGCCATGGTCCCGGCGGTAGGCCCATTGCTCGGAGCGCTCGTCGACATGTGGCTTGGGTGGCGGGCTATCTTTGCGTTTCTAGGTTTGGGCATGATCGCTGCATCTGCAGCAGCGTGGCGATTCTGGCCTGAAACCCGGGTGCAACGAGTTGCGGGCTTGCAATGGTCGCAGCTGCTACTCCCCGTTAAGTGCCTGAACTTCTGGTTGTACACGTTGTGTTACGCCGCTGGAATGGGTAGCTTCTTCGTCTTTTTCTCCATTGCGCCCGGACTAATGATGGGCAGGCAAGGTGTGTCTCAGCTTGGCTTCAGCCTGCTGTTCGCCACAGTGGCAATTGCCATGGTGTTTACGGCTCGTTTTATGGGGCGTGTGATACCCAAGTGGGGCAGCCCAAGTGTCTTGCGAATGGGAATGGGATGCCTGATAGCTGGAGCAGTATTGCTTGCCATCACCGAAATATGGGCTTTGCAGTCCGTGTTAGGCTTTATTGCTCCAATGTGGCTAGTGGGTATTGGTGTCGCCACAGCGGTATCTGTGGCGCCCAATGGCGCTCTTC | [CP083586](https://www.ncbi.nlm.nih.gov/nuccore/CP083586) | 100.00 |
|  | GN16 | CACCGCCCAAGCAGAAGTAGACTGCCGTGACCGTTCCAGCAACATGGTCGAATCCTCGAAGAGCGCCATTGGGCGACACAGATACCGCTGTGGCGACACCAATACCCACTAGCCACATTGGAGCAATAAAGCCTAACACGGACTGCGAAGCCCATATTTCGGTGATGGCAAGCAATACTGCTCCAGCTATCAGGCATCCCATTCCCATTCGCAAGACACTTGGGCTGCCCCACTTGGGTATCACACGCCCCATAAAACGAGCCGTAANNNNNNNNNNAACACCATGGCAATTGCCACTGTGGCGAACAGCAGGCTGAAGCCAAGCTGAGACACACCTTGCCTGCCCATCATTAGTCCGGGCGCAATGGAGAAAAAGACGAAGAAGCTACCCATTCCAGCGGCGTAACACAACGTGTACAACCAGAAGTTCAGGCACTTAACGGGGAGTAGCAGCTGCGACCATTGCAAGCCCGCAACTCGTTGCACCCGGGTTTCAGGCCAGAATCGCCACGCTGCTGCAGATGCAGCGATCATGCCCAAACCTAGAAACGCAAAGATAGCCCGCCACCCAAGCCACATGTCGACGAGCGCTCCGAGCAATGGGCCTACCGCCGGGACCATGGCCAGCATGGATCCGAGTATGCCGTAAATGACATTACTTTCCTCGCGACCTGCGTAAATGTCACGTACTGTTGCAAATGTGGAAACAAGGCACGCCGAGGCACCACAAGCCTGAAGAATCCGAAGCCCCAGAAAGACTTCAGCCGATGACGTAAGAGCGAGGCCCATTGACGCCACAACGTAGGCGAGGCCACCTCCCAGTAGAACGGGGCGGCGCCCCAGTCGGTCCGATAGCGGTCCAAACAAGAGCTGACCGGCACCAATCATGACCAAGTACGT | [CP038456](https://www.ncbi.nlm.nih.gov/nuccore/CP038456) | 98.78 |
|  | ZQ15 | TGCCAGCAGTGCCGTTTATGCCAAACGCGCTTGGTACGACAGCGAGCACAATTCAGCTTACGCTGACAACGTACTTGGTCATGATTGGTGCCGGTCAGCTCTTGTTTGGACCGCTATCGGACCGACTGGGGCGCCGCCCCGTTCTACTGGGAGGTGGCCTCGCCTACGTTGTGGCGTCAATGGGCCTCGCTCTTACGTCATCGGCTGAAGTCTTTCTGGGGCTTCGGATTCTTCAGGCTTGTGGTGCCTCGGCGTGCCTTGTTTCCACATTTGCAACAGTACGTGACATTTACGCAGGTCGCGAGGAAAGTAATGTCATTTACGGCATACTCGGATCCATGCTGGCCATGGTCCCGGCGGTAGGCCCATTGCTCGGAGCGCTCGTCGACATGTGGCTTGGGTGGCGGGCTATCTTTGCGTTTCTAGGTTTGGGCATGATCGCTGCATCTGCAGCAGCGTGGCGATTCTGGCCTGAAACCCGGGTGCAACGAGTTGCGGGCTTGCAATGGTCGCAGCTGCTACTCCCCGTTAAGTGCCTGAACTTCTGGTTGTACACGTTGTGTTACGCCGCTGGAATGGGTAGCTTCTTCGTCTTTTTCTCCATTGCGCCCGGACTAATGATGGGCAGGCAAGGTGTGTCTCAGCTTGGCTTCAGCCTGCTGTTCGCCACAGTGGCAATTGCCATGGTGTTTACGGCTCGTTTTATGGGGCGTGTGATACCCAAGTGGGGCAGCCCAAGTGTCTTGCGAATGGGAATGGGATGCCTGATAGCTGGAGCAGTATTGCTTGCCATCACCGAAATATGGGCTTCGCAGTCCGTGTTAGGCTTTATTGCTCCAATGTGGCTAGTGGGTATTGGTGTCGCCACAGCGGTATCTGTGTCGCCCAATGGCGCTCTTC | [CP038456](https://www.ncbi.nlm.nih.gov/nuccore/CP038456) | 100.00 |
|  | GN31 | TGCCAGCAGTGCCGTTTATGCCAAACGCGCTTGGTACGACAGCGAGCACAATTCAGCTTACGCTGACAACGTACTTGGTCATGATTGGTGCCGGTCAGCTCTTGTTTGGACCGCTATCGGACCGACTGGGGCGCCGCCCCGTTCTACTGGGAGGTGGCCTCGCCTACGTTGTGGCGTCAATGGGCCTCGCTCTTACGTCATCGGCTGAAGTCTTTCTGGGGCTTCGGATTCTTCAGGCTTGTGGTGCCTCGGCGTGCCTTGTTTCCACATTTGCAACAGTACGTGACATTTACGCAGGTCGCGAGGAAAGTAATGTCATTTACGGCATACTCGGATCCATGCTGGCCATGGTCCCGGCGGTAGGCCCATTGCTCGGAGCGCTCGTCGACATGTGGCTTGGGTGGCGGGCTATCTTTGCGTTTCTAGGTTTGGGCATGATCGCTGCATCTGCAGCAGCGTGGCGATTCTGGCCTGAAACCCGGGTGCAACGAGTTGCGGGCTTGCAATGGTCGCAGCTGCTACTCCCCGTTAAGTGCCTGAACTTCTGGTTGTACACGTTGTGTTACGCCGCTGGAATGGGTAGCTTCTTCGTCTTTTTCTCCATTGCGCCCGGACTAATGATGGGCAGGCAAGGTGTGTCTCAGCTTGGCTTCAGCCTGCTGTTCGCCACAGTGGCAATTGCCATGGTGTTTACGGCTCGTTTTATGGGGCGTGTGATACCCAAGTGGGGCAGCCCAAGTGTCTTGCGAATGGGAATGGGATGCCTGATAGCTGGAGCAGTATTGCTTGCCATCACCGAAATATGGGCTTCGCAGTCCGTGTTAGGCTTTATTGCTCCAATGTGGCTAGTGGGTATTGGTGTCGCCACAGCGGTATCTGTGTCGCCCAATGGCGCTCTTCGAGGATTCGAC | [CP038456](https://www.ncbi.nlm.nih.gov/nuccore/CP038456) | 100.00 |
| *floR* | dc308 | ATGACCACCACACGCCCCGCGTGGGCCTATACGCTGCCGGCAGCACTGCTGCTGATGGCTCCTTTCGACATCCTCGCTTCACTGGCGATGGATATTTATCTCCCTGTCGTTCCAGCGATGCCCGGCATCCTGAACACGACGCCCGCTATGATCCAACTCACGTTGAGCCTCTATATGGTGATGCTCGGCGTGGGCCAGGTGATTTTTGGTCCGCTCTCAGACAGAATCGGGCGACGGCCAATTCTACTTGCGGGCGCAACGGCTTTCGTCATTGCGTCTCTGGGAGCAGCTTGGTCTTCAACTGCACCGGCCTTTGTCGCTTTCCGTCTACTTCAAGCAGTGGGCGCGTCGGCCATGCTGGTGGCGACGTTCGCGACGGTTCGCGACGTTTATGCCAACCGTCCTGAGGGTGTCGTCATCTACGGCCTTTTCAGTTCGGTGCTGGCGTTCGTGCCTGCGCTCGGCCCTATCGCCGGAGCATGGATCGGCGAGTTCTTGGGATGGCAGGCGATATTCATTACTTTGGCTATACTGGCGATGCTCGCACTCCTAAATGCGGGTTTCAGGTGGCACGAAACCCGCCCTCTGGATCAAGTCAAGACGCGCCGATCTGTCTTGCCGATCTTCGCGAGTCCGGCTTTTTGGGTTTACACTGTCGGCTTTAGCGCCGGTATGGGCACCTACTTCGTCTTCTTCTCGACGGCTCCCCGTGTGCTCATAGGCCAAGCGGAATATTCCGAGATCGGATTCAGCTTTGCCTTCGCCACTGTCGCGCTTGTAATGATCGTGACAACCCGTTTCGCGAAGTCCTTTGTCGCCAGATGGGGCATCGCAGGATGCGTGGCGCGTGGGATGGCGTTGCTTGTTTGCGGAGCGGTCCTGTTGGGGATCGGCGAACTTTACGGCTCGCCGTCATTCCTCACCTTCATCCTACCGATGTGGGTTGTCGCGGTCGGTATTGTCTTCACGGTGTCCGTTACCGCGAACGGCGCTTTGGCAGAGTTCGACGACATCGCGGGATCAGCGGTCGCGTTCTACTTCTGCGTTCAAAGCCTGATAGTCAGCATTGTCGGGACATTGGCGGTGGCACTTTTAAACGGTGACACAGCGTGGCCCGTGATCTGTTACGCCACGGCGATGGCGGTACTGGTTTCGTTGGGGCTGGTGCTCCTTCGGCTCCGTGGGGCTGCCACCGAGAAGTCGCCAGTCGTCTAA | [CP053724](https://www.ncbi.nlm.nih.gov/nuccore/CP053724) | 100.00 |
|  | NMB6 | ATGACCACCACACGCCCCGCGTGGGCCTATACGCTGCCGGCAGCACTGCTGCTGATGGCTCCTTTCGACATCCTCGCTTCACTGGCGATGGATATTTATCTCCCTGTCGTTCCAGCGATGCCCGGCATCCTGAACACGACGCCCGCTATGATCCAACTCACGTTGAGCCTCTATATGGTGATGCTCGGCGTGGGCCAGGTGATTTTTGGTCCGCTCTCAGACAGAATCGGGCGACGGCCAATTCTACTTGCGGGCGCAACGGCTTTCGTCATTGCGTCTCTGGGAGCAGCTTGGTCTTCAACTGCACCGGCCTTTGTCGCTTTCCGTCTACTTCAAGCAGTGGGCGCGTCGGCCATGCTGGTGGCGACGTTCGCGACGGTTCGCGACGTTTATGCCAACCGTCCTGAGGGTGTCGTCATCTACGGCCTTTTCAGTTCGATGCTGGCGTTCGTGCCTGCGCTCGGCCCTATCGCCGGAGCATTGATCGGCGAGTTCTTGGGATGGCAGGCGATATTCATTACTTTGGCTATACTGGCGATGCTCGCACTCCTAAATGCGGGTTTCAGGTGGCACGAAACCCGCCCTCTGGATCAAGTCAAGACGCGCCGATCTGTCTTGCCGATCTTCGCGAGTCCGGCTTTTTGGGTTTACACTGTCGGCTTTAGCGCCGGTATGGGCACCTACTTCGTCTTCTTCTCGACGGCTACCCGTGTGCTCATAGGCCAAGCGGAATATTCCGAGATCGGATTCAGCTTTGCCTTCGCCACTGTCGCGCTTGTAATGATCGTGACAACCCGTTTCGCGAAGTCCTTTGTCGCCAGATGGGGCATCGCAGGATGCGTGGCGCGTGGGATGGCGTTGCTTGTTTGCGGAGCGGTCCTGTTGGGGATCGGCGAACTTTACGGCTCGCCGTCATTCCTCACCTTCATCCTACCGATGTGGGTTGTCGCGGTCGGTATTGTCTTCACGGTGTCCGTTACCGCGAACGGCGCTTTGGCAGAGTTCGACGACATCGCGGGATCAGCGGTCGCGTTCTACTTCTGCGTTCAAAGCCTGATAGTCAGCATTGTCGGGACATTGGCGGTGGCACTTTTAAACGGTGACACAGCGTGGCCCGTGATCTGTTACGCCACGGCGATGGCGGTACTGGTTTCGTTGGGGCTGGTGCTCCTTCGGCTCCGTGGGGCTGCCACCGAGAAGTCGCCAGTCGTCTAA | [CP055262](https://www.ncbi.nlm.nih.gov/nuccore/CP055262) | 100.00 |
|  | GN16 | TTAGACGACTGGCGACTTCTCGGTGGCAGCCCCACGGAGCCGAAGGAGCACCAGCCCCAACGAAACCAGTACCGCCATCGCCGTGGCGTAACAGATCACGGGCCACGCTGTGTCACCGTTTAAAAGTGCCACCGCCAATGTCCCGACAATGCTGACTATCAGGCTTTGAACGCAGAAGTAGAACGCGACCGCTGATCCCGCGATGTCGTCGAACTCTGCCAAAGCGCCGTTCGCGGTAACGGACACCGTGAAGACAATACCGACCGCGACAACCCACATCGGTAGGATGAAGGTGAGGAATGACGGCGAGCCGTAAAGTTCGCCGATCCCCAACAGGACCGCTCCGCAAACAAGCAACGCCATCCCACGCGCCACGCATCCTGCGATGCCCCATCTGGCGACAAAGGACTTCGCGAAACGGGTTGTCACGATCATTACAAGCGCGACAGTGGCGAAGGCAAAGCTGAATCCGATCTCGGAATATTCCGCTTGGCCTATGAGCACACGGGTAGCCGTCGAGAAGAAGACGAAGTAGGTGCCCATACCGGCGCTAAAGCCGACAGTGTAAACCCAAAAAGCCGGACTCGCGAAGATCGGCAAGACAGATCGGCGCGTCTTGACTTGATCCAGAGGGCGGGTTTCGTGCCACCTGAAACCCGCATTTAGGAGTGCGAGCATCGCCAGTATAGCCAAAGTAATGAATATCGCCTGCCATCCCAAGAACTCGCCGATCAATGCTCCGGCGATAGGGCCGAGCGCAGGCACGAACGCCAGCATCGAACTGAAAAGGCCGTAGATGACGACACCCTCAGGACGGTTGGCATAAACGTCGCGAACCGTCGCGAACGTCGCCACCAGCATGGCCGACGCGCCCACTGCTTGAAGTAGACGGAAAGCGACAAAGGCCGGTGCAGTTGAAGACCAAGCTGCTCCCAGAGACGCAATGACGAAAGCCGTTGCGCCCGCAAGTAGAATTGGCCGTCGCCCGATTCTGTCTGAGAGCGGACCAAAAATCACCTGGCCCACGCCGAGCATCACCATATAGAGGCTCAACGTGAGTTGGATCATAGCGGGCGTCGTGTTCAGGATGCCGGGCATCGCTGGAACGACAGGGAGATAAATATCCATCGCCAGTGAAGCGAGGATGTCGAAAGGAGCCATCAGCAGCAGTGCTGCCGGCAGCGTATAGGCCCACGCGGGGCGTGTGGTGGTCAT | [CP055262](https://www.ncbi.nlm.nih.gov/nuccore/CP055262) | 100.00 |
|  | F8 | TTAGACGACTGGCGACTTCTCGGTGGCAGCCCCACGGAGCCGAAGGAGCACCAGCCCCAACGAAACCAGTACCGCCATCGCCGTGGCGTAACAGATCACGGGCCACGCTGTGTCACCGTTTAAAAGTGCCACCGCCAATGTCCCGACAATGCTGACTATCAGGCTTTGAACGCAGAAGTAGAACGCGACCGCTGATCCCGCGATGTCGTCGAACTCTGCCAAAGCGCCGTTCGCGGTAACGGACACCGTGAAGACAATACCGACCGCGACAACCCACATCGGTAGGATGAAGGTGAGGAATGACGGCGAGCCGTAAAGTTCGCCGATCCCCAACAGGACCGCTCCGCAAACAAGCAACGCCATCCCACGCGCCACGCATCCTGCGATGCCCCATCTGGCGACAAAGGACTTCGCGAAACGGGTTGTCACGATCATTACAAGCGCGACAGTGGCGAAGGCAAAGCTGAATCCGATCTCGGAATATTCCGCTTGGCCTATGAGCACACGGGTAGCCGTCGAGAAGAAGACGAAGTAGGTGCCCATACCGGCGCTAAAGCCGACAGTGTAAACCCAAAAAGCCGGACTCGCGAAGATCGGCAAGACAGATCGGCGCGTCTTGACTTGATCCAGAGGGCGGGTTTCGTGCCACCTGAAACCCGCATTTAGGAGTGCGAGCATCGCCAGTATAGCCAAAGTAATGAATATCGCCTGCCATCCCAAGAACTCGCCGATCAATGCTCCGGCGATAGGGCCGAGCGCAGGCACGAACGCCAGCATCGAACTGAAAAGGCCGTAGATGACGACACCCTCAGGACGGTTGGCATAAACGTCGCGAACCGTCGCGAACGTCGCCACCAGCATGGCCGACGCGCCCACTGCTTGAAGTAGACGGAAAGCGACAAAGGCCGGTGCAGTTGAAGACCAAGCTGCTCCCAGAGACGCAATGACGAAAGCCGTTGCGCCCGCAAGTAGAATTGGCCGTCGCCCGATTCTGTCTGAGAGCGGACCAAAAATCACCTGGCCCACGCCGAGCATCACCATATAGAGGCTCAACGTGAGTTGGATCATAGCGGGCGTCGTGTTCAGGATGCCGGGCATCGCTGGAACGACAGGGAGATAAATATCCATCGCCAGTGAAGCGAGGATGTCGAAAGGAGCCATCAGCAGCAGTGCTGCCGGCAGCGTATAGGCCCACGCGGGGCGTGTGGTGGTCAT | [CP055262](https://www.ncbi.nlm.nih.gov/nuccore/CP055262) | 100.00 |
| *sul3* | GN5 | GTGGAGCATTTCCCAGCCTCACTAAAGCAGCAATTCTTTCTTTAAAAAATTCCATCATGGAAGTAAAAACCTCTTCCGGATTCGTTTCAACTTTAGTAGCTGCACCAATTCGCTGA | [CP084535](https://www.ncbi.nlm.nih.gov/nuccore/CP084535) | 99.05 |
|  | Z30 | TCGGGCTTACCCAGCCTCACTAAAGCAGCAATTCTTTCTTTAAAAAATTCCATCATGGAAGTAAAAACCTCTTCCGGATTCGTTTCAACTTTAGTAGCTGCACCAATTCGCTGA | [CP084535](https://www.ncbi.nlm.nih.gov/nuccore/CP084535) | 99.05 |
|  | L3 | GTTGGCTTTACCAGCCTCACTAAAGCAGCAATTCTTTCTTTAAAAAATTCCATCATGGAAGTAAAAACCTCTTCCGGATTCGTTTCAACTTTAGTAGCTGCACCAATTCGCTGA | [OK001321](https://www.ncbi.nlm.nih.gov/nuccore/OK001321) | 99.06 |
|  | dc308 | CCCATACCCGGATCAAGAATAATTCGTTCATGCTTTACACCAGCCTCAACTAAAGCAGCAATTCTTTCTTTAAAAAATTCCATCATGGAAGTAAAAACCTCTTCCGGATTCGTTTCAACTTTAGTAGCTGCACCAATTCGCTG | [OK001321](https://www.ncbi.nlm.nih.gov/nuccore/OK001321) | 99.30 |
|  | Z16 | CAGCGAATTGGTGCAGCTACTAAAGTTGAAACGAATCCGGAAGAGGTTTTTACTTCCATGATGGAATTTTTTAAAGAAAGAATTGCTGCTTTAGTTGAGGCTGGTGTAAAGCGTGAACGAATTATTCTTGATCCGGGTATGGG | [OK001321](https://www.ncbi.nlm.nih.gov/nuccore/OK001321) | 100.00 |
| *tet*(B) | GN3 | ACTCCCCTGAGCTTGAGGGGTTAACATGAAGGTCATCGATAGCAGGATAATAATACAGTAAAACGCTAAACCAATAATCCAAATCCAGCCATCCCAAATTGGTAGTGAATGATTATAAATAACAGCAAACAGTAATGGGCCAATAACACCGGTTGCATTGGTAAGGCTCACCAATAATCCCTGTAAAGCACCTTGCTGATGACTCTTTGTTTGGATAGACATCACTCCCTGTAATGCAGGTAAAGCGATCCCACCACCAGCCAATAAAATTAAAACAGGGAAAACTAACCAACCTTCAGATATAAACGCTAAAAAGGCAAATGCACTACTATCTGCAATAAATCCGAGCAGTACTGCCGTTTTTTCGCCCCATTTAGTGGCTATTCTTCCTGCCACAAAGGCTTGGAATACT | [CP084529](https://www.ncbi.nlm.nih.gov/nuccore/CP084529) | 100.00 |
|  | GN28 | ACTCCCCTGAGCTTGAGGGGTTAACATGAAGGTCATCGATAGCAGGATAATAATACAGTAAAACGCTAAACCAATAATCCAAATCCAGCCATCCCAAATTGGTAGTGAATGATTATAAATAACAGCAAACAGTAATGGGCCAATAACACCGGTTGCATTGGTAAGGCTCACCAATAATCCCTGTAAAGCACCTTGCTGATGACTCTTTGTTTGGATAGACATCACTCCCTGTAATGCAGGTAAAGCGATCCCACCACCAGCCAATAAAATTAAAACAGGGAAAACTAACCAACCTTCAGATATAAACGCTAAAAAGGCAAATGCACTACTATCTGCAATAAATCCGAGCAGTACTGCCGTTTTTTCGCCCCATTTAGTGGCTATTCTTCCTGCCACAAAGGCTTGGAATACT | [CP084529](https://www.ncbi.nlm.nih.gov/nuccore/CP084529) | 100.00 |
|  | dc70 | ACTCCCCTGAGCTTGAGGGGTTAACATGAAGGTCATCGATAGCAGGATAATAATACAGTAAAACGCTAAACCAATAATCCAAATCCAGCCATCCCAAATTGGTAGTGAATGATTATAAATAACAGCAAACAGTAATGGGCCAATAACACCGGTTGCATTGGTAAGGCTCACCAATAATCCCTGTAAAGCACCTTGCTGATGACTCTTTGTTTGGATAGACATCACTCCCTGTAATGCAGGTAAAGCGATCCCACCACCAGCCAATAAAATTAAAACAGGGAAAACTAACCAACCTTCAGATATAAACGCTAAAAAGGCAAATGCACTACTATCTGCAATAAATCCGAGCAGTACTGCCGTTTTTTCGCCCCATTTAGTGGCTATTCTTCCTGCCACAAAGGCTTGGAATACT | [CP084529](https://www.ncbi.nlm.nih.gov/nuccore/CP084529) | 100.00 |
|  | ZQ19 | CTCAGTATTCCAAGCCTTTGTGGCAGGAAGAATAGCCACTAAATGGGGCGAAAAAACGGCAGTACTGCTCGGATTTATTGCAGATAGTAGTGCATTTGCCTTTTTAGCGTTTATATCTGAAGGTTGGTTAGTTTTCCCTGTTTTAATTTTATTGGCTGGTGGTGGGATCGCTTTACCTGCATTACAGGGAGTGATGTCTATCCAAACAAAGAGTCATCAGCAAGGTGCTTTACAGGGATTATTGGTGAGCCTTACCAATGCAACCGGTGTTATTGGCCCATTACTGTTTGCTGTTATTTATAATCATTCACTACCAATTTGGGATGGCTGGACTTGGATTATTGGTTTAGCGTTTTACTGTATTATTATCCTGCTATCGATGACCTTCATGTTAACCCCTCAAGCTCAGGGGAGT | [CP084529](https://www.ncbi.nlm.nih.gov/nuccore/CP084529) | 99.76 |
| *tet*(M) | ZQ13 | AGATTCGGTAAAGTTCATTACACGCATATTAGGATGCAGTTCTACCTTCTGTTTGATTACAATTTCCGCAGAAAGTTTCTCTTTAATATCCTGATAAACCGTTGATAAATCAATTCCATTTTGGTCAATCTTATTGATAAAAAAGATTGTGGGAATACCTATTTTCCTAAGTGCATGAAACAATATACGAGTTTGTGCTTGTACGCCATCTTTTGCAGAAATCAGTAGAATTGCCCCATCTAATACTGATAATGAACGATATACTTCTGCTAAGAAATCCATATGTCCTGGCGTGTCTATGATGTTCACCTTCGTATTTTCCCACTGAAAAGAGGTTATTCCTGTCTGAATTGTAATTCCTCTCTGACGTTCTAAAAGCGTATTATCCGTCCTCGTTGTACCTTTGTCCAC | [CP053737](https://www.ncbi.nlm.nih.gov/nuccore/CP053737) | 100.00 |
|  | GN4 | AGATTCGGTAAAGTTCATTACACGCATATTAGGATGCAGTTCTACCTTCTGTTTGATTACAATTTCCGCAGAAAGTTTCTCTTTAATATCCTGATAAACCGTTGATAAATCAATTCCATTTTGGTCAATCTTATTGATAAAAAAGATTGTGGGAATACCTATTTTCCTAAGTGCATGAAACAATATACGAGTTTGTGCTTGTACGCCATCTTTTGCAGAAATCAGTAGAATTGCCCCATCTAATACTGATAATGAACGATATACTTCTGCTAAGAAATCCATATGTCCTGGCGTGTCTATGATGTTCACCTTCGTATTTTCCCACTGAAAAGAGGTTATTCCTGTCTGAATTGTAATTCCTCTCTGACGTTCTAAAAGCGTATTATCCGTCCTCGTTGTACCTTTGTCCAC | [CP053737](https://www.ncbi.nlm.nih.gov/nuccore/CP053737) | 100.00 |
|  | GN5 | CAGATTCGGTAAAGTTCATTACACGCATATTAGGATGCAGTTCTACCTTCTGTTTGATTACAATTTCCGCAGAAAGTTTCTCTTTAATATCCTGATAAACCGTTGATAAATCAATTCCATTTTGGTCAATCTTATTGATAAAAAAGATTGTGGGAATACCTATTTTCCTAAGTGCATGAAACAATATACGAGTTTGTGCTTGTACGCCATCTTTTGCAGAAATCAGTAGAATTGCCCCATCTAATACTGATAATGAACGATATACTTCTGCTAAGAAATCCATATGTCCTGGCGTGTCTATGATGTTCACCTTCGTATTTTCCCACTGAAAAGAGGTTATTCCTGTCTGAATTGTAATTCCTCTCTGACGTTCTAAAAGCGTATTATCCGTCCTCGTTGTACCTTTGTCCAC | [CP053729](https://www.ncbi.nlm.nih.gov/nuccore/CP053729) | 100.00 |
|  | dc70 | CAGATTCGGTAAAGTTCATTACACGCATATTAGGATGCAGTTCTACCTTCTGTTTGATTACAATTTCCGCAGAAAGTTTCTCTTTAATATCCTGATAAACCGTTGATAAATCAATTCCATTTTGGTCAATCTTATTGATAAAAAAGATTGTGGGAATACCTATTTTCCTAAGTGCATGAAACAATATACGAGTTTGTGCTTGTACGCCATCTTTTGCAGAAATCAGTAGAATTGCCCCATCTAATACTGATAATGAACGATATACTTCTGCTAAGAAATCCATATGTCCTGGCGTGTCTATGATGTTCACCTTCGTATTTTCCCACTGAAAAGAGGTTATTCCTGTCTGAATTGTAATTCCTCTCTGACGTTCTAAAAGCGTATTATCCGTCCTCGTTGTACCTTTGTCCAC | [CP053722](https://www.ncbi.nlm.nih.gov/nuccore/CP053722) | 100.00 |
|  | dc308 | CGGTAAAGTTCGTCACACACATATTAGGATACAGTTCTACCTTCTGTTTGATTACAATTTCGGCAGAAAGTTTCTCTTTAATATCCTGATAAACCGTTGATAAATCAATTCCATTTTGGTCAATCTTATTGATAAAAAAGATTGTGGGAATCCCCATTTTCCTAAGTGCATGAAATAATATACGAGTTTGTGCTTGTACGCCATCTTTTGCAGAAATCAGTAGAATTGCCCCATCTAAAACTGATAATGAACGATATACTTCTGCTAAGAAATCCATATGTCCTGGCGTGTCTATGATGTTCACCTTCGTATTTTCCCACTGAAAAGAGGTTATTCCTGTCTGAATTGTAATTCCTCTCTGACGTTCTAAAAGCGTATTATCCGTCCTCGTTGTACCTTTGTCCAC | [CP084535](https://www.ncbi.nlm.nih.gov/nuccore/CP084535) | 100.00 |
| *sul1* | P42 | AAGTTCCGCCGCAAGGCTCGCTGGACCCAGATCCTTTACAGGAAGGCCAACGGTGGCGCCCAAGAAGGATTTCCGCGACACCGAGACCAATAGCGGAAGCCCCAACGCCGACTTCAGCTTTTGAAGGTTCGACAGCACGTGCAGCGATGTTTCCGGTG | [CP082828](https://www.ncbi.nlm.nih.gov/nuccore/CP082828) | 100.00 |
|  | ZQ27 | CACCGGAAACATCGCTGCACGTGCTGTCGAACCTTCAAAAGCTGAAGTCGGCGTTGGGGCTTCCGCTATTGGTCTCGGTGTCGCGGAAATCCTTCTTGGGCGCCACCGTTGGCCTTCCTGTAAAGGATCTGGGTCCAGCGAGCCTTGCGGCGGAACTT | [CP082828](https://www.ncbi.nlm.nih.gov/nuccore/CP082828) | 100.00 |
|  | GN49 | AAGTTCCGCCGCAAGGCTCGCTGGACCCAGATCCTTTACAGGAAGGCCAACGGTGGCGCCCAAGAAGGATTTCCGCGACACCGAGACCAATAGCGGAAGCCCCAACGCCGACTTCAGCTTTTGAAGGTTCGACAGCACGTGCAGCGATGTTTCCGGTG | [CP082828](https://www.ncbi.nlm.nih.gov/nuccore/CP082828) | 100.00 |
|  | ZQ23 | CACCGGAAACATCGCTGCACGTGCTGTCGAACCTTCAAAAGCTGAAGTCGGCGTTGGGGCTTCCGCTATTGGTCTCGGTGTCGCGGAAATCCTTCTTGGGCGCCACCGTTGGCCTTCCTGTAAAGGATCTGGGTCCAGCGAGCCTTGCGGCGGAACTT | [CP082828](https://www.ncbi.nlm.nih.gov/nuccore/CP082828) | 100.00 |
|  | D12 | AAGTTCCGCCGCAAGGCTCGCTGGACCCAGATCCTTTACAGGAAGGCCAACGGTGGCGCCCAAGAAGGATTTCCGCGACACCGAGACCAATAGCGGAAGCCCCAACGCCGACTTCAGCTTTTGAAGGTTCGACAGCACGTGCAGCGATGTTTCCGGTG | [CP082828](https://www.ncbi.nlm.nih.gov/nuccore/CP082828) | 100.00 |
| *sul2* | P42 | CTCCGATGGAGGCCGGTATCTGGCGCCAGACGCAGCCATTGCGCAGGCGCGTAAGCTGATGGCCGAGGGGGCAGATGTGATCGACCTCGGTCCGGCATCCAGCAATCCCGACGCCGCGCCTGTTTCGTCCGACACAGAAATCGCGCGTATCGCGCCGGTGCTGGACGCGCTCAAGGCAGATGGCATTCCC | [CP066845](https://www.ncbi.nlm.nih.gov/nuccore/CP066845) | 100.00 |
|  | GN49 | CTCCGATGGAGGCCGGTATCTGGCGCCAGACGCAGCCATTGCGCAGGCGCGTAAGCTGATGGCCGAGGGGGCAGATGTGATCGACCTCGGTCCGGCATCCAGCAATCCCGACGCCGCGCCTGTTTCGTCCGACACAGAAATCGCGCGTATCGCGCCGGTGCTGGACGCGCTCAAGGCAGATGGCATTCCC | [CP084535](https://www.ncbi.nlm.nih.gov/nuccore/CP084535) | 100.00 |
|  | dc308 | GGGAATGCCATCTGCCTTGAGCGCGTCCAGCACCGGCGCGATACGCGCGATTTCTGTGTCGGACGAAACAGGCGCGGCGTCGGGATTGCTGGATGCCGGACCGAGGTCGATCACATCTGCCCCCTCGGCCATCAGCTTACGCGCCTGCGCAATGGCTGCGTCTGGCGCCAGATACCGGCCTCCATCGGAG | [CP084534](https://www.ncbi.nlm.nih.gov/nuccore/CP084534) | 100.00 |
|  | NMB6 | GGGAATGCCATCTGCCTTGAGCGCGTCCAGCACCGGCGCGATACGCGCGATTTCTGTGTCGGACGAAACAGGCGCGGCGTCGGGATTGCTGGATGCCGGACCGAGGTCGATCACATCTGCCCCCTCGGCCATCAGCTTACGCGCCTGCGCAATGGCTGCGTCTGGCGCCAGATACCGGCCTCCATCGGAG | [CP066845](https://www.ncbi.nlm.nih.gov/nuccore/CP066845) | 100.00 |
|  | ZQ19 | GGGAATGCCATCTGCCTTGAGCGCGTCCAGCACCGGCGCGATACGCGCGATTTCTGTGTCGGACGAAACAGGCGCGGCGTCGGGATTGCTGGATGCCGGACCGAGGTCGATCACATCTGCCCCCTCGGCCATCAGCTTACGCGCCTGCGCAATGGCTGCGTCTGGCGCCAGATACCGGCCTCCATCGGAG | [CP066845](https://www.ncbi.nlm.nih.gov/nuccore/CP066845) | 100.00 |
| *intI1* | ZQ19 | GAAAGGTCTGGTCATACATGTGATGGCGACGCACGACACCGCTCCGTGGATCGGTCGAATGCGTGTGCTGCGCAAAAACCCAGAACCACGGCCAGGAATGCCCGGCGCGCGGATACTTCCGCTCAAGGGCGTCGGGAAGCGCAACGCCGCTGCGGCCCTCGGCCTGGTCCTTCAGCCACCATGCCCGTGCACGCGACAGCTGCTCGCGCAGGCTGGGTGCCAAGCTCTCGGGTAACATCAAGGCCCGATCCTTGGAGCCCTTGCCCTCCCGCACGATGATCGTGCCGTGATCGAAATCCAGATCCTTGACCCGCAGTTGCAAACCCTCACTGATCCGCATGCCCGTTCCATACAGAAGCTGGGCGAACAAACGATGCTCGCCTTCCAGAAAACCGAGGATGCGAACCACTTCATCCGGGGTCAGCACCACCGGCAAGCGCCGCGACGGCCGAGGTCTTCCGATCTCCTGAAGCCAGGGCAGATCCGTGCACAGCACCTTGCCGTAGAAGAACAGCAAGGCCGCCAATGCCTGACGATGCGTGGAGACCGAAACCTTGCGCTCGT | [CP066845](https://www.ncbi.nlm.nih.gov/nuccore/CP066845) | 100.00 |
|  | NMB6 | GAAAGGTCTGGTCATACATGTGATGGCGACGCACGACACCGCTCCGTGGATCGGTCGAATGCGTGTGCTGCGCAAAAACCCAGAACCACGGCCAGGAATGCCCGGCGCGCGGATACTTCCGCTCAAGGGCGTCGGGAAGCGCAACGCCGCTGCGGCCCTCGGCCTGGTCCTTCAGCCACCATGCCCGTGCACGCGACAGCTGCTCGCGCAGGCTGGGTGCCAAGCTCTCGGGTAACATCAAGGCCCGATCCTTGGAGCCCTTGCCCTCCCGCACGATGATCGTGCCGTGATCGAAATCCAGATCCTTGACCCGCAGTTGCAAACCCTCACTGATCCGCATGCCCGTTCCATACAGAAGCTGGGCGAACAAACGATGCTCGCCTTCCAGAAAACCGAGGATGCGAACCACTTCATCCGGGGTCAGCACCACCGGCAAGCGCCGCGACGGCCGAGGTCTTCCGATCTCCTGAAGCCAGGGCAGATCCGTGCACAGCACCTTGCCGTAGAAGAACAGCAAGGCCGCCAATGCCTGACGATGCGTGGAGACCGAAACCTTGCGCTCGT | [CP084537](https://www.ncbi.nlm.nih.gov/nuccore/CP084537) | 100.00 |
|  | NMJ7 | ACGAGCGCAAGGTTTCGGTCTCCACGCATCGTCAGGCATTGGCGGCCTTGCTGTTCTTCTACGGCAAGGTGCTGTGCACGGATCTGCCCTGGCTTCAGGAGATCGGAAGACCTCGGCCGTCGCGGCGCTTGCCGGTGGTGCTGACCCCGGATGAAGTGGTTCGCATCCTCGGTTTTCTGGAAGGCGAGCATCGTTTGTTCGCCCAGCTTCTGTATGGAACGGGCATGCGGATCAGTGAGGGTTTGCAACTGCGGGTCAAGGATCTGGATTTCGATCACGGCACGATCATCGTGCGGGAGGGCAAGGGCTCCAAGGATCGGGCCTTGATGTTACCCGAGAGCTTGGCACCCAGCCTGCGCGAGCAGCTGTCGCGTGCACGGGCATGGTGGCTGAAGGACCAGGCCGAGGGCCGCAGCGGCGTTGCGCTTCCCGACGCCCTTGAGCGGAAGTATCCGCGCGCCGGGCATTCCTGGCCGTGGTTCTGGGTTTTTGCGCAGCACACGCATTCGACCGATCCACGGAGCGGTGTCGTGCGTCGCCATCACATGTATGACCAGACCTTTC | [CP084535](https://www.ncbi.nlm.nih.gov/nuccore/CP084535) | 100.00 |
|  | S10 | ACGAGCGCAAGGTTTCGGTCTCCACGCATCGTCAGGCATTGGCGGCCTTGCTGTTCTTCTACGGCAAGGTGCTGTGCACGGATCTGCCCTGGCTTCAGGAGATCGGAAGACCTCGGCCGTCGCGGCGCTTGCCGGTGGTGCTGACCCCGGATGAAGTGGTTCGCATCCTCGGTTTTCTGGAAGGCGAGCATCGTTTGTTCGCCCAGCTTCTGTATGGAACGGGCATGCGGATCAGTGAGGGTTTGCAACTGCGGGTCAAGGATCTGGATTTCGATCACGGCACGATCATCGTGCGGGAGGGCAAGGGCTCCAAGGATCGGGCCTTGATGTTACCCGAGAGCTTGGCACCCAGCCTGCGCGAGCAGCTGTCGCGTGCACGGGCATGGTGGCTGAAGGACCAGGCCGAGGGCCGCAGCGGCGTTGCGCTTCCCGACGCCCTTGAGCGGAAGTATCCGCGCGCCGGGCATTCCTGGCCGTGGTTCTGGGTTTTTGCGCAGCACACGCATTCGACCGATCCACGGAGCGGTGTCGTGCGTCGCCATCACATGTATGACCAGACCTTTC | [CP066845](https://www.ncbi.nlm.nih.gov/nuccore/CP066845) | 100.00 |
|  | GN46 | GAAAGGTCTGGTCATACATGTGATGGCGACGCACGACACCGCTCCGTGGATCGGTCGAATGCGTGTGCTGCGCAAAAACCCAGAACCACGGCCAGGAATGCCCGGCGCGCGGATACTTCCGCTCAAGGGCGTCGGGAAGCGCAACGCCGCTGCGGCCCTCGGCCTGGTCCTTCAGCCACCATGCCCGTGCACGCGACAGCTGCTCGCGCAGGCTGGGTGCCAAGCTCTCGGGTAACATCAAGGCCCGATCCTTGGAGCCCTTGCCCTCCCGCACGATGATCGTGCCGTGATCGAAATCCAGATCCTTGACCCGCAGTTGCAAACCCTCACTGATCCGCATGCCCGTTCCATACAGAAGCTGGGCGAACAAACGATGCTCGCCTTCCAGAAAACCGAGGATGCGAACCACTTCATCCGGGGTCAGCACCACCGGCAAGCGCCGCGACGGCCGAGGTCTTCCGATCTCCTGAAGCCAGGGCAGATCCGTGCACAGCACCTTGCCGTAGAAGAACAGCAAGGCCGCCAATGCCTGACGATGCGTGGAGACCGAAACCTTGCGCTCGT | [CP066845](https://www.ncbi.nlm.nih.gov/nuccore/CP066845) | 100.00 |
